# Estimation in meta-analyses of response ratios

**DOI:** 10.1186/s12874-020-01137-1

**Published:** 2020-10-22

**Authors:** Ilyas Bakbergenuly, David C. Hoaglin, Elena Kulinskaya

**Affiliations:** 1 School of Computing Sciences, University of East Anglia, Norwich Research Park, Norwich, NR4 7TJ UK; 2 Department of Population and Quantitative Health Sciences, University of Massachusetts Medical School, 368 Plantation Street, Worcester, 01605 Massachusetts USA

**Keywords:** Between-study variance, Heterogeneity, Random-effects model, Meta-analysis, Log-response-ratio, Ratio of means

## Abstract

**Background:**

For outcomes that studies report as the means in the treatment and control groups, some medical applications and nearly half of meta-analyses in ecology express the effect as the ratio of means (RoM), also called the response ratio (RR), analyzed in the logarithmic scale as the log-response-ratio, LRR.

**Methods:**

In random-effects meta-analysis of LRR, with normal and lognormal data, we studied the performance of estimators of the between-study variance, *τ*^2^, (measured by bias and coverage) in assessing heterogeneity of study-level effects, and also the performance of related estimators of the overall effect in the log scale, *λ*. We obtained additional empirical evidence from two examples.

**Results:**

The results of our extensive simulations showed several challenges in using LRR as an effect measure. Point estimators of *τ*^2^ had considerable bias or were unreliable, and interval estimators of *τ*^2^ seldom had the intended 95% coverage for small to moderate-sized samples (*n*<40). Results for estimating *λ* differed between lognormal and normal data.

**Conclusions:**

For lognormal data, we can recommend only SSW, a weighted average in which a study’s weight is proportional to its effective sample size, (when *n*≥40) and its companion interval (when *n*≥10). Normal data posed greater challenges. When the means were far enough from 0 (more than one standard deviation, 4 in our simulations), SSW was practically unbiased, and its companion interval was the only option.

## Background

Users of meta-analysis assemble estimated effects from several studies in order to assess their heterogeneity and obtain an overall estimate. Here we focus on the measure of effect known as the response ratio (RR, also known in medical applications as the ratio of means, RoM), analyzed in the logarithmic scale as the log-response-ratio, LRR. In ecology almost half of all meta-analyses use this outcome measure [1, 2]. When some studies report means but include no information on the corresponding variances, the alternative of analyzing the standardized mean difference is not available.

The RR was introduced by Hedges et al. [3] and rediscovered as RoM by Friedrich et al. [4]; they assumed normality of the underlying data. To avoid confusing RR with relative risk, we use RoM on the original scale and LRR on the log-scale. Because the LRR is not defined for negative values of the study means, Lajeunesse [5] modeled the data by lognormal distributions. We explore meta-analysis of LRR under both normal and lognormal distributions, combined with the fixed-effect model and the random-effects model.

The fixed-effect (FE) model regards the studies as sharing a single true effect, whereas the random-effects (RE) model regards those true effects as a sample from a distribution. The variance of that distribution, usually denoted by *τ*^2^, is often used in estimating the overall effect (the mean of the distribution of random effects). In studying estimation for meta-analysis of LRR, we focus first on *τ*^2^ and then proceed to the overall effect, which we denote by *λ*.

Veroniki et al. [6] give a broad overview of methods of estimating *τ*^2^ and its uncertainty. However, they study only “methods that can be applied for any type of outcome data.” As we show elsewhere [7], the performance of the methods varies widely among effect measures. Also, empirical information on the comparative performance of the methods remains limited. We address both of these limitations for the effect measure LRR.

Veroniki et al. [6, (Appendix Table 1)] cite no previous simulation studies on the comparative performance of estimators of *τ*^2^ for LRR.

Several studies have considered the quality of estimation of *λ*. Friedrich et al. [4] report extensive simulations for LRR under normality, but they use only the DerSimonian-Laird (DL) method to estimate *τ*^2^ and do not report on its quality. Lajeunesse [5] discusses bias correction for study-level LRR and its variance, and provides some simulation results for lognormal distributions, but there is no evidence on applicability of this correction under the RE model. Doncaster and Spake [8] provide some limited simulation results under normality for accuracy of estimation of the overall LRR and its variance, using the DL and restricted maximum-likelihood (REML) methods to estimate *τ*^2^. To assess bias of the estimators of *λ*, they use mean absolute error, which is not a measure of bias; it is the linear counterpart of mean squared error.

To address this gap in information on methods of estimating the heterogeneity variance for LRR, we use simulation to study four general-purpose methods recommended by Veroniki et al. [6]. These are the well-established methods of DerSimonian and Laird [9], restricted maximum likelihood, and Mandel and Paule [10] (MP) and the less-familiar method of Jackson [11]. We also study coverage of confidence intervals for *τ*^2^ achieved by four methods: the Q-profile method of Viechtbauer [12], the methods of Biggerstaff and Jackson [13] and Jackson [11], and the profile-likelihood-based interval [14].

For each estimator of *τ*^2^, we also study bias of the corresponding inverse-variance-weighted estimator of the overall effect. However, it is well known that these inverse-variance-weighted estimators have unacceptable bias for some other effect measures, as Bakbergenuly et al. [7] and Hamman et al. [15] show for the standardized mean difference and Bakbergenuly et al. [16] show for the log-odds-ratio. As a way of reducing this bias, we added an estimator (SSW) whose weights depend only on the sample sizes of the Treatment and Control arms. We study the coverage of the confidence intervals associated with the inverse-variance-weighted estimators, and also the HKSJ interval [17, 18], a modification of the HKSJ interval that uses the MP estimator of *τ*^2^ instead of the DL estimator, and an interval centered at SSW that uses the MP estimator of *τ*^2^ in estimating its variance and bases its width on a *t* distribution.

Under “Methods” we briefly review study-level estimation of log-response-ratio and the standard random-effects model for meta-analysis, discuss models for meta-analysis of log-response-ratio, very briefly discuss point and interval estimation of between-study variance and overall effect, introduce the SSW estimator, and describe our simulations. Then we summarize results of the simulations, discuss the two examples, and provide discussion and conclusions.

Additional files list the methods and provide additional plots and our R programs.

## Methods

### Study-level estimation of log-response-ratio

We assume that each of the *K* studies in the meta-analysis consists of two arms, Treatment and Control, with sample sizes *n*_*iT*_ and *n*_*iC*_. The total sample size in Study *i* is *n*_*i*_=*n*_*iT*_+*n*_*iC*_. The subject-level data in each arm are assumed to be normally or log-normally distributed with means *μ*_*iT*_ and *μ*_*iC*_ and variances $\sigma _{iT}^{2}$ and $\sigma _{iC}^{2}$. The sample means are $\bar {X}_{ij}$, and the sample variances are $s^{2}_{ij}$, for *i*=1,…,*K* and *j*=*C* or *T*.

The response ratio is usually meta-analyzed on a log scale, where the effect measure is *λ*_*i*_= log(*μ*_*iT*_/*μ*_*iC*_), estimated by $\hat \lambda _{i}=\log \left (\bar {X}_{iT}/\bar {X}_{iC}\right)$, and the population and sample means are assumed to be positive. As Bakbergenuly et al. [19] discuss, however, the log transformation introduces bias: the expected value of $\exp \left (\hat {\lambda }_{i}\right)$ is not equal to exp(*λ*_*i*_). The within-study variance estimate for $\hat {\lambda }_{i}$, obtained by the delta method, is [3]

1 $$ \hat{v}_{i}^{2}=\frac{s_{iT}^{2}}{n_{iT}\bar{X}_{iT}^{2}}+\frac{s_{iC}^{2}}{n_{iC}\bar{X}_{iC}^{2}}= \frac{\hat{V}_{iT}^{2}}{n_{iT}}+ \frac{\hat{V}_{iC}^{2}}{n_{iC}},  $$

where $\hat {V}_{ij}$ is the sample coefficient of variation (CV). The positively skewed distribution of $\hat {\lambda }_{i}$ is not well approximated by a normal distribution when at least one of the sample means is close to zero. This behavior results in considerable bias of $\hat {\lambda }_{i}$ and affects the quality of its variance estimate [3]. The standard advice is to use LRR for Study *i* only when at least one of $\sqrt {n_{ij}} \bar {X}_{ij} / s_{ij} \geq 3$. This advice is often ignored in practice [5].

To eliminate this bias in small samples, Lajeunesse [5] proposed two bias-corrected modifications, and he recommended

2 $$  \hat\lambda^{\Delta}_{i}=\hat\lambda_{i}+\frac{1}{2}\left [ \frac{s_{iT}^{2}}{n_{iT}\bar{X}_{iT}^{2}}-\frac{s_{iC}^{2}}{n_{iC}\bar{X}_{iC}^{2}}\right ]  $$

and estimated its variance by

3 $$  \widehat{\text{Var}}\left(\hat\lambda^{\Delta}_{i}\right)=v_{i}^{2}+\frac{1}{2}\left [ \frac{s_{iT}^{4}}{n_{iT}^{2}\bar{X}_{iT}^{4}}-\frac{s_{iC}^{4}}{n_{iC}^{2}\bar{X}_{iC}^{4}}\right].  $$

However, this bias-corrected estimator and its variance would be even less stable for small values of $\bar {X}_{ij}$.

### Standard random-effects model

The standard random-effects model accounts for within- and between-study variabilities by assuming approximately normal distributions of within- and between-study effects. We use this model to facilitate comparison with previous work, which has relied on it, and because applications have used methods based on it. This traditional model, however, has shortcomings. For most measures of effect, within-study variability is only approximately normal, generally in large samples. In practice, between-study variability is unlikely to be normal, but few meta-analyses involve enough studies to reliably evaluate that assumption or to point toward suitable alternatives. However, some simulation evidence [20] indicates that various methods based on the random-effects model are largely robust to departures from normality.

For a generic measure of effect, denoted by *λ*,

4 $$ \hat{\lambda}_{i} \sim N\left(\lambda_{i}, {v}_{i}^{2}\right) \quad \text{and} \quad \lambda_{i} \sim N\left(\lambda,\tau^{2}\right),  $$

the resulting marginal distribution is $\hat {\lambda }_{i} \sim N\left (\lambda, v_{i}^{2}+\tau ^{2}\right)$. $\hat {\lambda }_{i}$ is the estimate of the effect in study *i*, and its within-study variance is $v_{i}^{2}$, estimated by $\hat {v}_{i}^{2}, i=1,\ldots, K$. The between-study variance, *τ*^2^, is estimated by $\hat {\tau }^{2}$. The overall effect, *λ*, is customarily estimated by the weighted mean

5 $$ \hat{\lambda}_{\mathit{RE}}=\frac{\sum\limits_{i=1}^{K}\hat{w}_{i}\left(\hat{\tau}^{2}\right)\hat{\lambda}_{i}}{\sum\limits_{i=1}^{K}\hat{w}_{i}\left(\hat{\tau}^{2}\right)}  $$

with inverse-variance (IV) weights $\hat {w}_{i}\left (\hat {\tau }^{2}\right)=\left (\hat {v}_{i}^{2}+\hat {\tau }^{2}\right)^{-1}$. In the FE estimate $\hat {\lambda }=\hat \lambda _{FE}$ the weights are $\hat {w}_{i} = \hat {w}_{i}(0)$. A reviewer raised the concern that, by using IV weights, random-effects estimators of the overall effect redistribute weight from larger studies (which contain more information) to smaller studies. Whenever $\hat {\tau }^{2} > 0$, such redistribution is an inherent feature of IV weights. Under “Point estimators” we discuss an estimator that avoids this shortcoming.

If the $\text {Var}(\hat {\lambda }_{i})$ are the (known) true variances and $w_{i} = 1 / \text {Var}(\hat {\lambda }_{i})$, the variance of the weighted mean of the $\hat {\lambda }_{i}$ is $1/ \sum w_{i}$. Thus, many authors estimate the variance of $\hat {\lambda }_{\mathit {RE}}$ by $\left [\sum _{i=1}^{K}\hat {w}_{i}\left (\hat {\tau }^{2}\right)\right ]^{-1}$. In practice, however, this estimator may not be satisfactory [21–23].

Several methods of estimating *τ*^2^ use Cochran’s *Q* statistic [24]

6 $$  Q = \sum \limits_{i=1}^{K} \hat{w}_{i}(0) \left(\hat{\lambda}_{i} - \hat{\lambda}\right)^{2}  $$

or a modification of it.

### Models for meta-analysis of log-response-ratio

Since $\hat {\lambda }$ is defined only in the positive quadrant $\left (\bar {X}_{T}>0,\;\bar {X}_{C}>0\right)$, the standard FE and RE models of meta-analysis are not quite appropriate for normally distributed data. (We exclude the extraneous and unlikely possibility $\bar {X}_{T} < 0,\;\bar {X}_{C} < 0$.) Let $\bar {X}_{j}\sim N\left (\mu _{j},\sigma _{j}^{2}/n_{j}\right)$. Then, under the fixed-effect model, the probability that $\hat {\lambda }$ is not defined is

$${}\begin{aligned} 1&-P\left(\bar{X}_{T}>0\right)P\left(\bar{X}_{C}>0\right)\\&= 1 - \left[1 - \Phi\left(-\sqrt{n_{C}}\mu_{C} / \sigma_{C}\right)\right] \left[1 - \Phi\left(-\sqrt{n_{T}}\mu_{T} /\sigma_{T}\right)\right], \end{aligned} $$

where *Φ*(·) is the cumulative distribution function of the standard normal distribution. This probability is a decreasing function of *n*_*j*_ and an increasing function of *V*_*j*_=*σ*_*j*_/*μ*_*j*_, and it is close to 0 for large sample sizes. However, very small sample sizes, starting from *n*_*T*_=*n*_*C*_=2, are abundant in ecology, where the LRR is a measure of choice. Keeping, say, $\sqrt {n_{C}}/V_{C}=3$, the probability that $\hat {\lambda }$ is not defined is 0.0027 for $\sqrt {n_{T}}/V_{T}=3$, 0.024 for $\sqrt {n_{T}}/V_{T}=2$, 0.160 for $\sqrt {n_{T}}/V_{T}=1$, and 0.309 for $\sqrt {n_{T}}/V_{T}=0.5$. Therefore, only large values of both $\sqrt {n_{C}}/V_{C}$ and $\sqrt {n_{T}}/V_{T}$ would avoid problems. When the population value $\sqrt {n_{T}}/V_{T}<3$, say, studies for which $\hat {\lambda }$ is not defined would occur with comparatively high probability and would need to be dropped from the meta-analysis. These omissions imply that the mean in each arm comes from a normal distribution truncated from below at $\sqrt {n_{j}}/V_{j}$, even under the FE model. This truncation may introduce bias in $\hat {\lambda }$.

Restrictions on the range are not unique to LRR; they apply to other effect measures such as the relative risk [25], where the restriction arises because the probabilities of events must be between 0 and 1.

In the RE model, *λ*_*i*_∼*N*(*λ*,*τ*^2^) and can be negative. If so, *μ*_*iT*_ (=*μ*_*iC*_ exp(*λ*_*i*_)) may be close to 0, and consequently the contribution of $\hat {V}_{iT}^{2} / n_{iT}$ to $\hat {v}_{i}^{2}$ in Eq. (1) may be large, further increasing the probability of truncation for $\bar {X}_{iT}$.

Because $\hat {\lambda }$ is not defined for negative values of the study means, Lajeunesse [5] modeled the data by lognormal distributions. In principle, lognormal distributions often make sense for non-negative data. This choice would eliminate the restricted-range bias of LRR. Of course, the choice of model should be based on the properties of the data and not on perceived ease of statistical modeling.

Even though sample means and variances are unbiased estimators of the population means and variances for lognormal distributions, they are very inefficient, especially in variance estimation [26, Section 14.4.1, p. 220–222]. If the data are assumed to come from lognormal distributions, a much more straightforward approach would log-transform the individual observations, which would reduce the problem to meta-analysis of mean difference. This approach would provide much better inferences. However, when individual-level data are not available, meta-analyses must work with the sample means and variances.

### Methods of estimating between-study variance

In this section we briefly list point and interval estimators of between-study variance *τ*^2^ used in our study.

#### Point estimators

The most popular (but rather biased) estimator of *τ*^2^ is the method-of-moments estimator of DerSimonian and Laird [9] (DL), denoted by $\hat {\tau }_{\mathit {DL}}^{2}$. It is based on the appoximate first moment of Cochran’s *Q*, Eq. (6).

Assuming that the $\hat {\lambda }_{i}$ are distributed as $N\left (\lambda,\hat {v}_{i}^{2}+\tau ^{2}\right)$, the restricted-maximum-likelihood (REML) estimator $\hat {\tau }_{\mathit {REML}}^{2}$ maximizes the restricted (or residual) log-likelihood function *l*_*R*_(*λ*,*τ*^2^). REML is superior to DL because of its balance between unbiasedness and efficiency [27].

DerSimonian and Kacker [28] generalized DL, replacing the weights $\hat {w}_{i}$ of Cochran’s *Q*, Eq. (6), by arbitrary fixed positive constants, *a*_*i*_. For situations in which some unknown amount of heterogeneity is anticipated, Jackson [11] proposed the estimator of *τ*^2^ with $a_{i} = 1 / \hat {v}_{i}$. We refer to this method as J.

The Mandel-Paule (MP) estimator [10], $\hat {\tau }_{\mathit {MP}}^{2}$, is another moment-based estimator of the between-study variance. Its calculation requires iteration. It is known to be superior to DL [6], but no simulations for LRR had been performed previously.

#### Interval estimators

The 95% profile-likelihood (PL) confidence interval consists of the values of *τ*^2^ that are not rejected by the likelihood-ratio test with *τ*^2^ as the null hypothesis [14]. This interval is usually used with $\hat {\tau }_{REML}^{2}$.

Similarly, the Q-profile confidence interval [12] consists of the values that are not rejected by the test based on a *Q* statistic (6) in which the weights are $\hat {w}_{i}\left (\tau ^{2}\right)$. The distribution of *Q* is assumed to follow the chi-square distribution with *K*−1 degrees of freedom.

For a generic effect measure, Biggerstaff and Jackson [13] derived the exact distribution of the *Q* statistic with arbitrary but constant weights *a*_*i*_. That distribution yielded a generalized Q-profile confidence interval. We refer to this interval with $a_{i}=\hat w_{i}$ as the BJ confidence interval.

Jackson [11] proposed another generalized Q-profile confidence interval (J). The approach is the same as for the BJ interval, but with $a_{i} = 1 /\hat {v}_{i}$.

### Methods of estimating overall effect

Because some of the point estimators of the overall effect do not have corresponding interval estimators, we describe point estimators and interval estimators in separate sections.

#### Point estimators

A random-effects method that estimates *λ* by a weighted mean with inverse-variance weights, as in Eq. (5), is determined by the particular $\hat {\tau }^{2}$ that it uses in $\hat {w}_{i}\left (\hat {\tau }^{2}\right)$. We refer to these estimators by the names of the respective $\hat {\tau }^{2}$. Because the study-level effects and their variances are related (see Eq. (1) for LRR), all inverse-variance-weighted estimators of overall LRR have considerable biases.

Following negative experience with the bias of inverse-variance-weighted estimators of SMD, Bakbergenuly et al. [7] included a point estimator whose weights depend only on the studies’ sample sizes, as proposed by Hedges and Olkin [29] and Hunter and Schmidt [30]. For this estimator (SSW), $w_{i} = \tilde {n}_{i} = n_{iT}n_{iC}/\left (n_{iT} + n_{iC}\right)$; that is, *w*_*i*_ sets $\hat {V}_{iT} = \hat {V}_{iC} = 1$ in Eq. (1). To reduce bias in estimating the overall LRR, we included SSW.

#### Interval estimators

The point estimators DL, REML, MP, and J have companion interval estimators of *λ*. The customary approach estimates the variance of $\hat {\lambda }_{\mathit {RE}}$ by $\left [\sum \limits _{i=1}^{K}\hat {w}_{i}\left (\hat {\tau }^{2}\right)\right ]^{-1}$ and bases the width of the interval on the normal distribution. These intervals are usually too narrow, and their coverage may also be reduced by bias. Hartung and Knapp [17] and, independently, Sidik and Jonkman [18] developed an improved estimator for the variance of $\hat {\lambda }_{\mathit {RE}}$. The Hartung-Knapp-Sidik-Jonkman (HKSJ) confidence interval uses this estimator and bases the width on critical values from the *t* distribution on *K*−1 degrees of freedom. A potential weakness of an HKSJ interval that uses $\hat {\lambda }_{\mathit {DL}}$ as its midpoint is that it will have any bias that is present in $\hat {\lambda }_{\mathit {DL}}$. Therefore we also consider an HKSJ confidence interval centered at $\hat \lambda _{MP}$ (HKSJ MP).

The interval estimator corresponding to SSW (SSW MP) uses the SSW point estimator as its center, and its width equals the estimated standard deviation of SSW under the random-effects model times twice the critical value from the *t* distribution on *K*−1 degrees of freedom. The estimator of the variance of SSW is [7, 5.2]

7 $$ \widehat{\text{Var}}\left(\hat{\lambda}_{\mathit{SSW}}\right)= \frac{\sum \tilde{n}_{i}^{2} \left(\hat{v}_{i}^{2} + \hat{\tau}^{2}\right)} {\left(\sum \tilde{n}_{i}\right)^{2}},  $$

in which $\hat {v}_{i}^{2}$ comes from Eq. (1) and $\hat {\tau }^{2} = \hat {\tau }_{\mathit {MP}}^{2}$.

### Simulation study

As mentioned under “Background”, a few studies have used simulation to examine estimators of the overall effect for LRR, but no studies have systematically examined estimators of *τ*^2^.

The range of values of LRR may be rather wide. The empirical study by Senior [31] reports values of LRR up to 3.72 (though the second-largest value is 1.46) and values of *K* from 3 to 592. The simulations by Friedrich et al. [4] used values of LRR up to 0.445 (RoM=1.56). Lajeunesse [5] used means between 0 and 8 in both arms and small sample sizes, starting from *n*_*T*_+*n*_*C*_=4. Unfortunately, no information is available on the accompanying range of *τ*^2^ values. In their simulations for SMD, Hamman et al. [15] consider the range from 0 to 2.5 as typical for ecology.

#### Design of the simulations

As summaries of performance our simulation study estimated bias of point estimators and coverage of interval estimators, for *τ*^2^ and for *λ*. Two basic distributions served as the source of the data in the Treatment and Control arms: the normal distribution (the subject of Bakbergenuly et al. [32]) and the lognormal distribution (the subject of a separate report [33]).

For the overall value of LRR, we chose *λ*=(0,0.2,0.5,1,2) (corresponding to 1≤RoM≤7.39), as realistic for a range of applications. The true values of LRR in the individual studies, *λ*_*i*_, were generated from a normal distribution: *λ*_*i*_∼*N*(*λ*,*τ*^2^). For a given Control mean *μ*_*iC*_, the Treatment mean was *μ*_*iT*_=*μ*_*iC*_ exp(*λ*_*i*_).

To evaluate performance issues related to proximity to zero, we used two values of the mean in the Control arm, *μ*_*iC*_=1 and *μ*_*iC*_=4, in simulations from normal distributions (with $\sigma ^{2}_{C} = 1$). When the data are lognormal, proximity to zero does not affect data generation or inferences. Therefore, as the mean of the Control arm we took *μ*_*iC*_=1.

All simulations used the same numbers of studies, small (*K*=5, 10, 30) and large (*K*=50, 100, 125), and equal numbers of observations in the Control and Treatment arms.

We studied only meta-analyses in which the study size was the same in all *K* studies and *n*_*iC*_=*n*_*iT*_=*n*_*i*_/2. The study sizes, *n*_*i*_, started from 4, because some studies in ecology have such small sample sizes; the small sample sizes were *n*_*i*_=4,10,20,40, and the large sample sizes were *n*_*i*_=100,250,640,1000. By using the same set of sample sizes for each combination of the other parameters, we avoided the additional variability in the results that would arise from choosing sample sizes at random (e.g., uniformly between 100 and 250).

For simulations from normal distributions, we generated the within-study sample variances $s_{ij}^{2}$ (*j*=*T*, *C*) from chi-square distributions as $\sigma _{ij}^{2}\chi _{n_{ij}-1}^{2}/(n_{ij}-1)$. We generated the estimated means $\bar {X}_{ij}$ from a normal distribution with mean *μ*_*ij*_ and variance $\sigma _{ij}^{2}/n_{ij}$. We obtained the estimated within-study LRR as $\hat {\lambda }_{i}=\log \left (\bar {X}_{iT}/\bar {X}_{iC}\right)$ and the estimated within-study variance from Eq. (1). Studies with at least one negative sample mean were discarded, and the value of *K* was reduced accordingly, resulting effectively in a simulation from a truncated normal distribution of means in each arm. The median number of studies was 4.859 (quartiles 4.612 and 4.961) for *K*=5, 9.715 (9.202, 9.925) for *K*=10, and 29.15 (27.60, 29.77) for *K*=30. In summary, we varied five parameters: the overall true LRR (*λ*), the between-studies variance (*τ*^2^), the mean in the Control arm (*μ*_*C*_), the number of studies (*K*), and the total sample size (*n*). We set $\sigma ^{2}_{C}=\sigma ^{2}_{T}=1$. Table 1 lists the configurations.

**Table 1:** Data patterns in the simulations for LRR

Parameter	Values	Full results in e-prints, appendices
*K* (number of studies: small/large)	(5, 10, 30) & (50, 100, 125)	A & B - small *n*
*n* (total study size: small/large)	(4, 10, 20, 40) & (100, 250, 640, 1000)	
$\sigma _{T}^{2}$ & $\sigma _{C}^{2}$ (within-study variances)	1 & 1	C & D - large *n*
*λ* (overall value of the LRR)	0, 0.2, 0.5, 1, 2	
*τ*^2^ (between-study variance)	0(0.1)1	
Normal distribution		Bakbergenuly et al. [32]
*μ*_*C*_ (mean in Control arm)	1, 4	appendices
estimation of *τ*^2^		A & C
estimation of *λ*		B & D
Lognormal distribution		Bakbergenuly et al. [33]
*μ*_*C*_ (mean in Control arm)	1	appendices
estimation of *τ*^2^		A & C
estimation of *λ*		B & D

For simulations from lognormal distributions, we generated *n*_*ij*_ independent observations from the lognormal distribution with mean $\log \left (\mu _{ij}\right)-0.5\log \left (1+\sigma _{ij}^{2}/\mu _{ij}^{2}\right)$ and variance $\log \left (1+\sigma _{ij}^{2}/\mu _{ij}^{2}\right)$, as in Lajeunesse [5], and obtained their sample means $\bar X_{ij}$ and sample variances $s^{2}_{ij}$. Then we calculated the sample LRR $\hat {\lambda }_{i}=\log \left (\bar {X}_{iT}/\bar {X}_{iC}\right)$ and the estimated variances $ \hat {v}_{i}^{2}$ as in Eq. (1). We also calculated the bias-corrected estimate, $\hat {\lambda }_{i}^{\Delta }$, Eq. (2), and its estimated variance, Eq. (3) [5]. In summary, we varied four parameters: the overall true LRR (*λ*), the between-studies variance (*τ*^2^), the number of studies (*K*), and the total sample size (*n*). We set $\sigma ^{2}_{C}=\sigma ^{2}_{T}=1$. Table 1 lists the configurations.

For each combination of parameters, we used a total of 10,000 repetitions. The resulting simulation standard error for estimated coverage of *τ*^2^ or *λ* at the 95% confidence level was roughly $\sqrt {0.95 \times 0.05 / 10,000} = 0.00218$.

We programmed the simulations in R version 3.3.2 using the University of East Anglia 140-computer-node High Performance Computing (HPC) Cluster, which has a total of 2560 CPU cores, including parallel processing and large memory resources. For each configuration, the 10,000 replications consisted of 10 parallel sets of 1000 replications.

A reviewer inquired about the values of *I*^2^ underlying our simulations. Figures A3 and A4 in Additional File 3 plot *I*^2^=100*τ*^2^/(*τ*^2^+*s*^2^) versus *τ*^2^∈[0,1] for LRR when *n* = 20, 40, 100, and 250, with traces for *λ* = 0, 0.5, 1, 1.5, and 2. As indicated by the definition, *I*^2^ increases as *τ*^2^ increases. The value of *n* also has a substantial impact (larger *n* yields higher *I*^2^). Importantly, for LRR *I*^2^ increases as *λ* increases, especially for the smaller *n*, contrary to the scale-invariance criterion of Higgins and Thompson [34]. We emphasize that we discourage use of *I*^2^, for reasons that include those mentioned here.

## Results

**Fig. 1:**
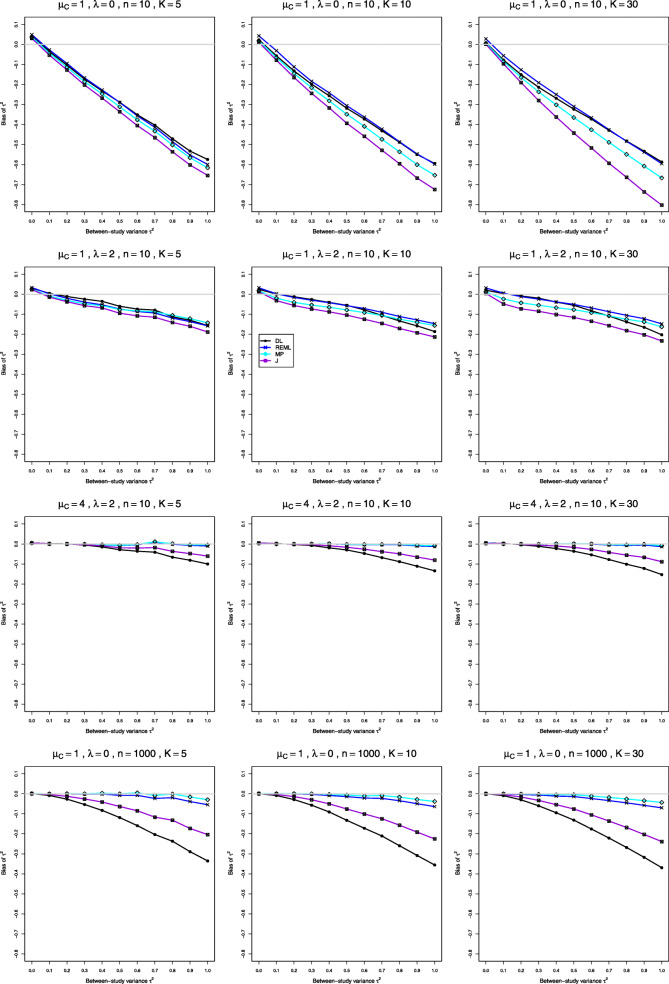
Bias in point estimators of between-study variance of LRR in simulations from normal distributions

**Fig. 2:**
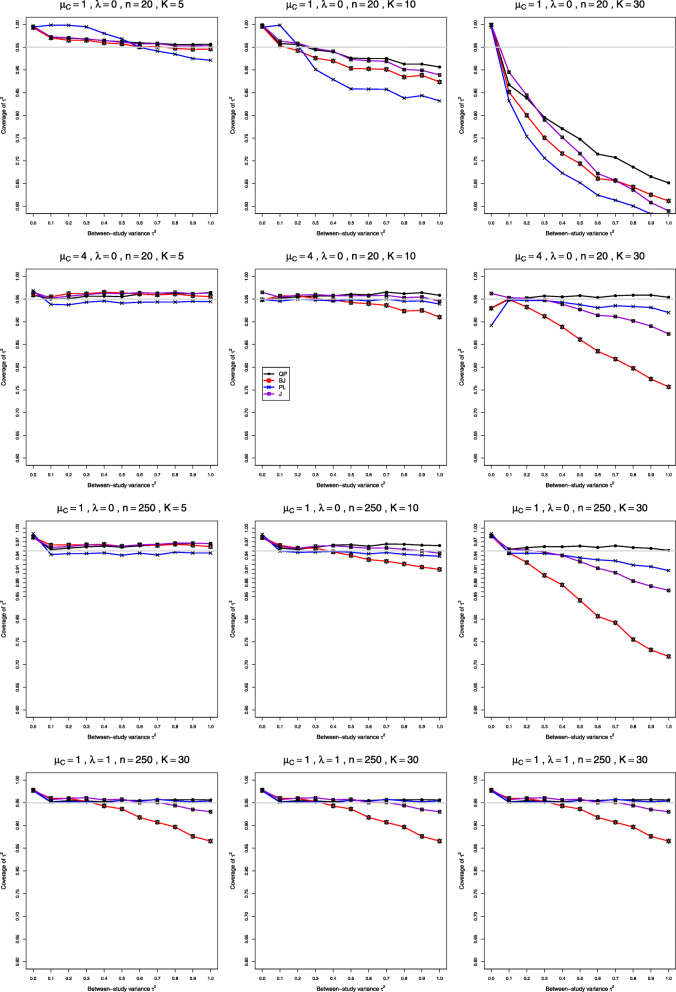
Coverage of 95% confidence intervals for between-study variance of LRR in simulations from normal distributions

**Fig. 3:**
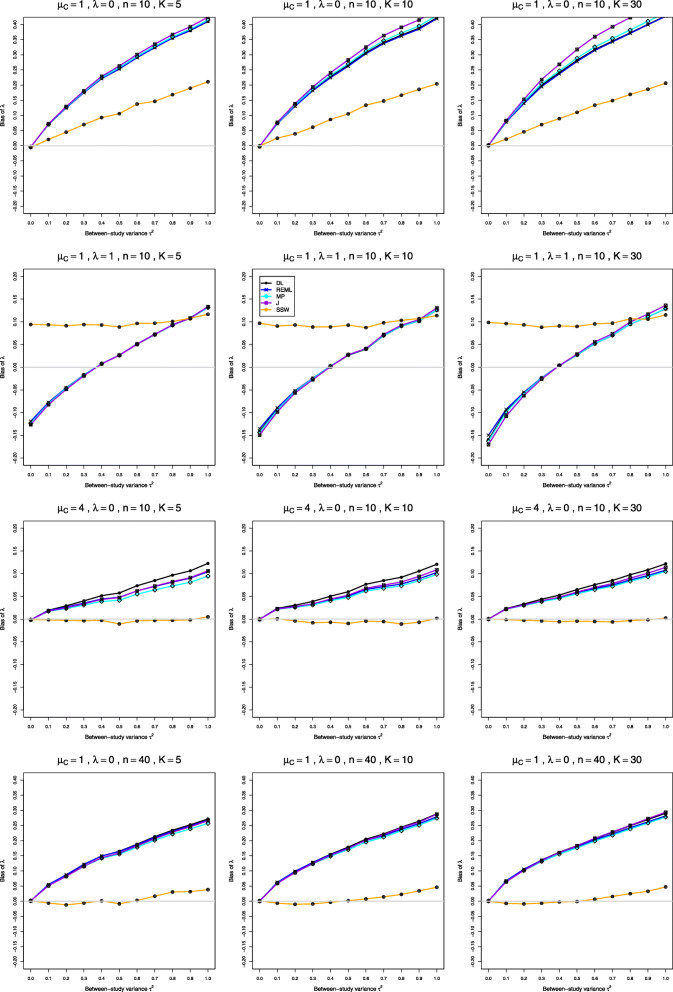
Bias in point estimators of *λ* in simulations from normal distributions

**Fig. 4:**
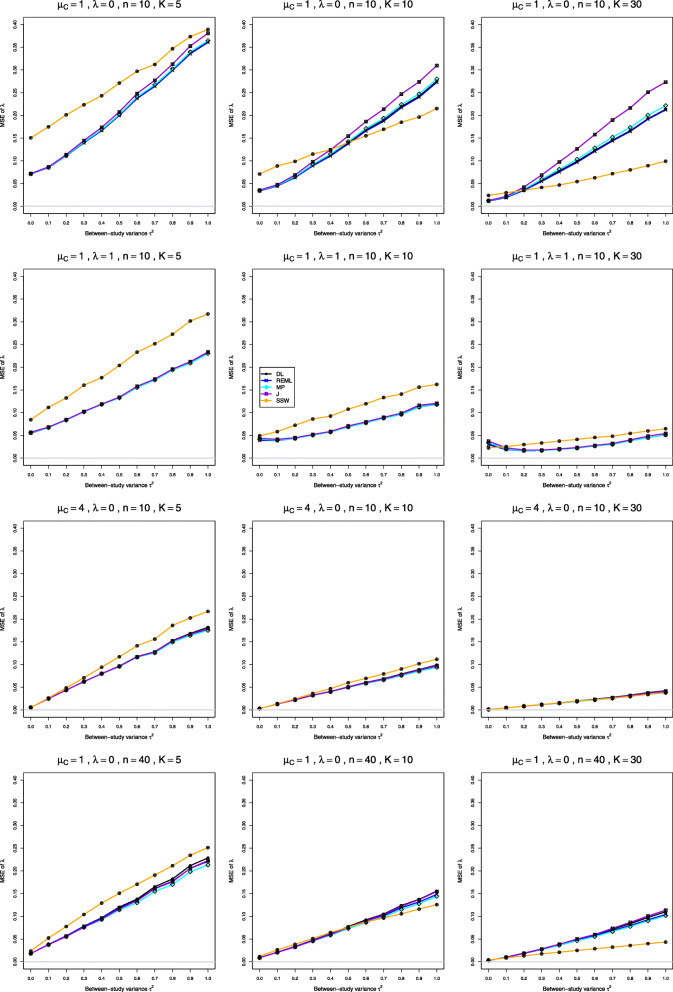
MSE of point estimators of *λ* in simulations from normal distributions

**Fig. 5:**
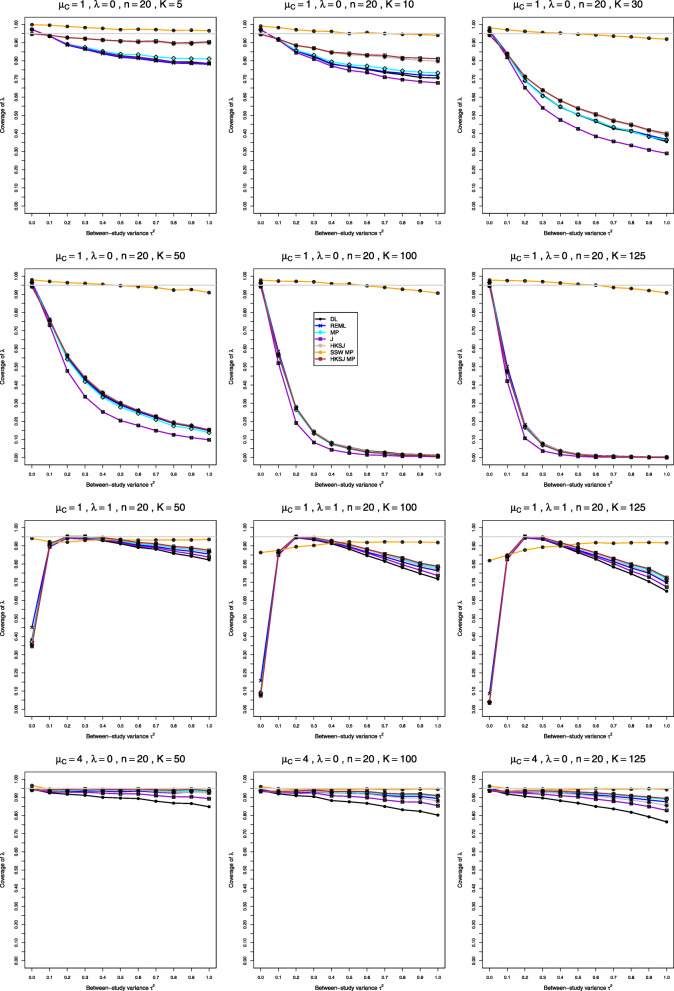
Coverage of 95% confidence intervals for *λ* in simulations from normal distributions

**Fig. 6:**
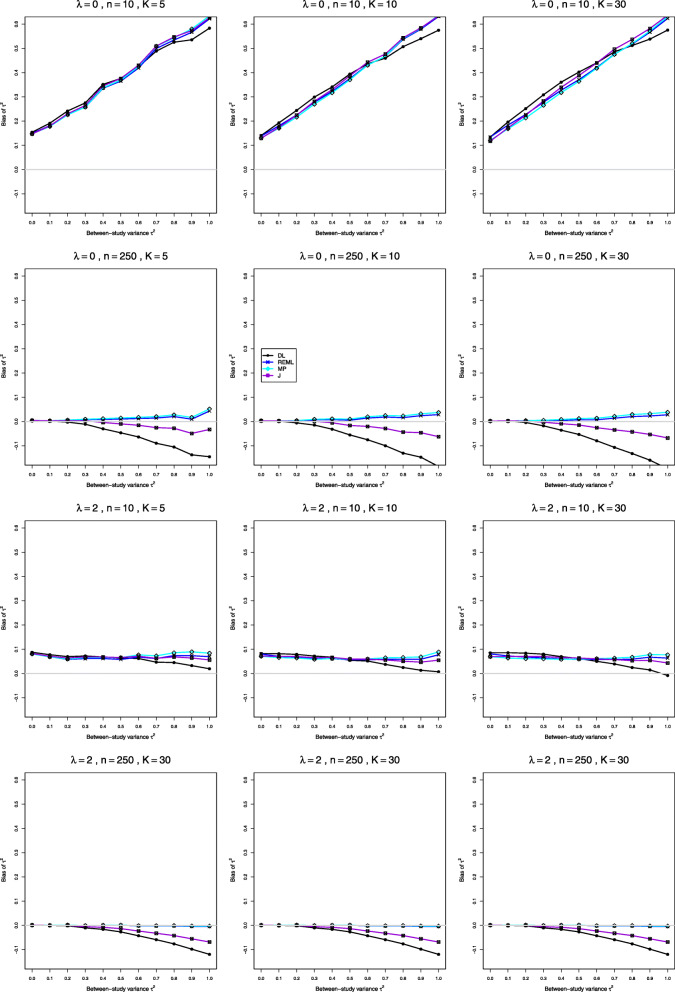
Bias in point estimators of between-study variance of LRR in simulations from lognormal distributions

**Fig. 7:**
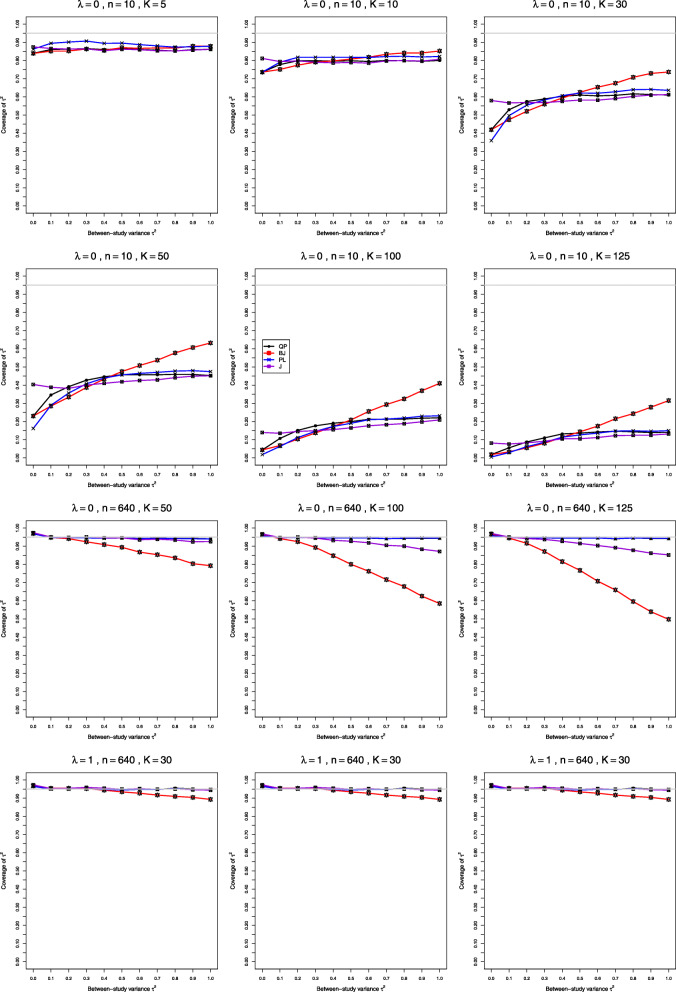
Coverage of 95% confidence intervals for between-study variance of LRR in simulations from lognormal distributions

**Fig. 8:**
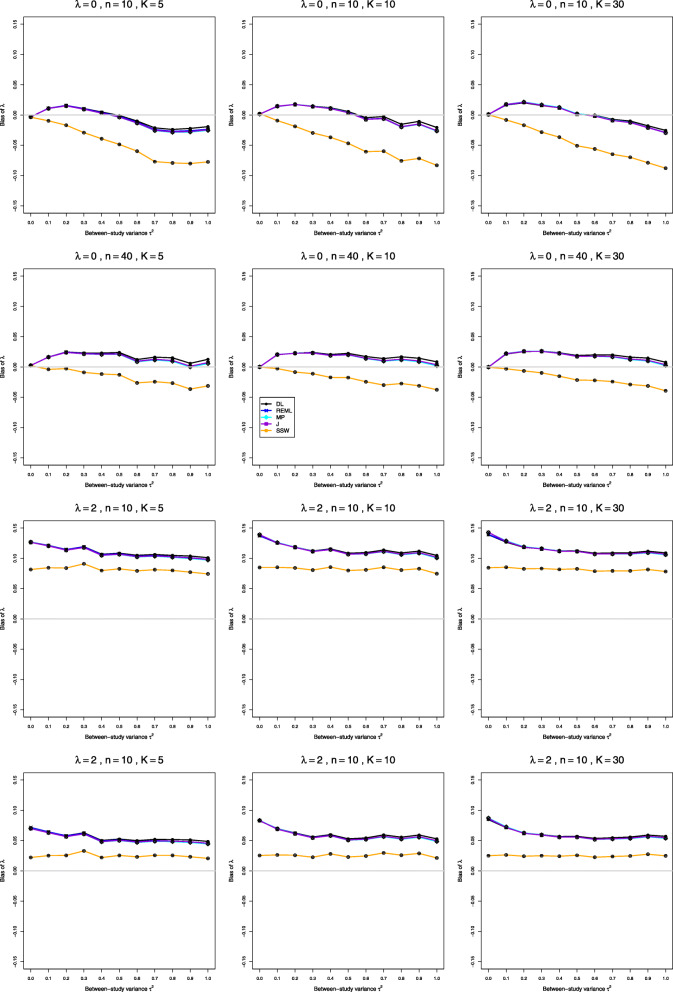
Bias in point estimators of *λ* in simulations from lognormal distributions. No bias correction in the first three rows; for comparison, bias-corrected estimation of *λ*_*i*_ in the fourth row

**Fig. 9:**
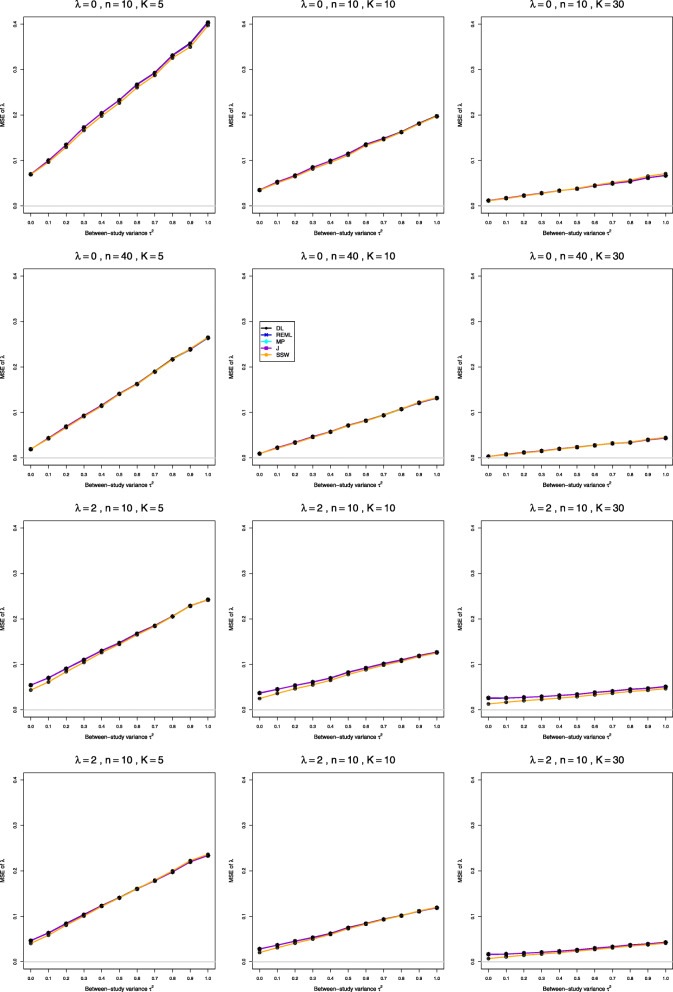
MSE of point estimators of *λ* in simulations from lognormal distributions. No bias correction in the first three rows; for comparison, bias-corrected estimation of *λ*_*i*_ in the fourth row

**Fig. 10:**
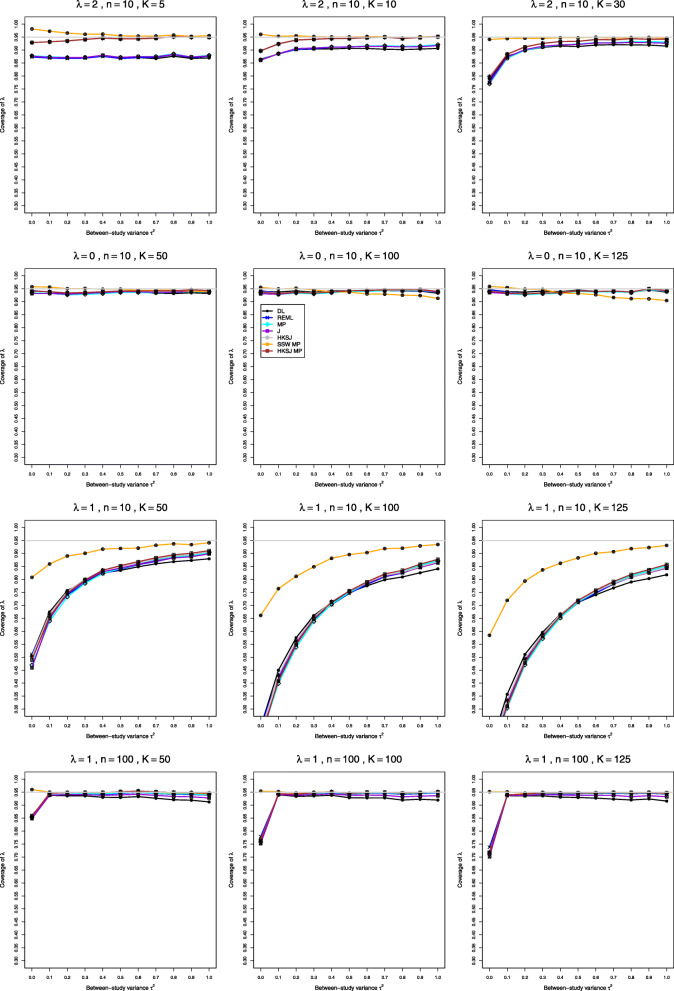
Coverage of 95% confidence intervals for *λ* in simulations from lognormal distributions. No bias correction in the first three rows; for comparison, bias-corrected estimation of *λ*_*i*_ in the fourth row

We report our full simulation results under the normal and lognormal distributions in e-prints [32, 33]. Those reports include 240 and 160 figures, respectively; each figure presents 12 plots (versus *τ*^2^) of bias or coverage of estimators of *τ*^2^ or estimators of *λ*, corresponding to four values of *n* and three values of *K*. The summary below is illustrated by Figs. 1, 2, 3, 4, 5, 6, 7, 8, 9 and 10. Those figures, however, do not give a comprehensive picture. In summarizing the results, we sometimes have to describe the joint contribution of two or three parameters.

### Bias and coverage in estimation of *τ*^2^ under normal distribution (Figs. 1 and 2)

**Bias** The main feature is a negative relation between bias and *τ*^2^. When *μ*_*C*_=1 and *n*=4, all four estimators have definite positive bias at *τ*^2^=0 (e.g., 0.13 to 0.22 when *λ*=0 and *K*=5). As *τ*^2^ increases, the bias trends downward, roughly linearly, and it can reach substantial negative values (e.g., −0.47 to −0.64 at *τ*^2^=1 when *λ*=0 and *K*=5). When *n*=10, the bias at *τ*^2^=0 is less than 0.05, and it goes to 0 as *n* increases. Also, the slopes of the traces decrease in magnitude, and the traces separate (Fig. 1, row 1). The separation is slight at *n*=40 but substantial at *n*=100. Interestingly, MP and REML improve steadily, but DL and J do not (Fig. 1, row 4); when *λ*=0,*K*=5, and *n*=1000, the bias at *τ*^2^=1 is −0.33 for DL and −0.20 for J versus −0.06 for REML and −0.03 for MP. Increasing *K* (even to 50, 100, and 125) generally does not reduce the biases, but the traces separate when *n*≤20, especially when *n*=4. As *λ* increases, the biases at *τ*^2^=0 decrease slightly, and the magnitudes of the slopes decrease substantially (Fig. 1, row 2); when *λ*=2 and *n*≥40, MP and REML are nearly unbiased. The biases are much smaller when *μ*_*C*_=4 (see Fig. 1, row 3 for comparison); we believe the difference is due to restricted-range issues. None of the four estimators of *τ*^2^ has adequately small bias overall, but (excluding *n*=4, which is generally quite challenging) MP often has the least bias, followed by REML, and J and DL have the most bias.

Bias increases when the bias-corrected estimator $\hat {\lambda }^{\Delta }$ is used for small *n*. As expected, bias-correction does not have any effect for large *n*.

**Coverage** When *μ*_*C*_=1, coverage of *τ*^2^ depends strongly on *K*. Counterintuitively, all four methods have coverage close to nominal when *K*=5, somewhat below nominal when *K*=10 (unless *n*>100), and often far below nominal when *K*≥30 (in problematic patterns when *n*=4). At *τ*^2^=0 coverage is noticeably above nominal when *n*≥10 (Fig. 2, row 1). When *n*≤40, coverage generally improves as *λ* increases (but it deteriorates at small *τ*^2^ when *n*=4). When *n*≥100, QP and PL generally have coverage reasonably close to nominal, even for *K*=30, and QP is sometimes closer; when *K*=30, the coverage of BJ and J decreases sharply as *τ*^2^ increases (except when *λ*=2) (Fig. 2, row 3). When *μ*_*C*_=4, coverage of the Q-profile intervals is close to nominal for *τ*^2^≥0.1 (Fig. 2, row 2). All four methods have below-nominal coverage at *τ*^2^=0 when *n*=4 and *λ*=0, and the deficit becomes worse and affects other values of *n* as *λ* increases. When *n* is large or *K* is large, the coverage of J and especially BJ declines steeply as *τ*^2^ increases (Fig. 2, row 4). Use of the bias-corrected estimator $\hat {\lambda }^{\Delta }$ does not affect coverage of *τ*^2^ for smaller *K* and makes it considerably worse for larger *K*.

### Bias, mean squared error, and coverage in estimation of *λ* under normal distribution (Figs. 3, 4 and 5)

**Bias and mean squared error** All four IV-weighted estimators of *λ* have practically the same bias. When *μ*_*C*_=1 and *λ*=0, the bias is 0 at *τ*^2^=0 and increases as *τ*^2^ increases (the traces resemble $\sqrt {\tau ^{2}}$ for *τ*^2^≤0.4 and are nearly linear for *τ*^2^≥0.4) (Fig. 3, row 1). At *τ*^2^=1 it can be large when *n* is small (e.g., around 0.40 when *K*=5 and *n*≤20). When *λ*≥0.2, the bias is negative at small *τ*^2^, with magnitude exceeding 0.12 when *λ*≥1 and *n*≤10; it becomes positive as *τ*^2^ increases. The bias can be large for small sample sizes, and it decreases to zero only for very large sample sizes. The bias depends very little on *K*. The bias of SSW is positive, though considerably less than the bias of the IV-weighted methods, and it decreases much more rapidly as *n* increases (Fig. 3, row 4). It decreases for increasing values of *λ*, staying at about 5% for *n*=10 and *λ*=2. Interestingly, when *λ*≥1 and *n*≥10, the bias of SSW (when positive) is roughly constant across the range of *τ*^2^ (Fig. 3, row 2). The bias of the IV-weighted estimators is still present, though much smaller, when *μ*_*C*_=4, whereas SSW is practically unbiased (Fig. 3, row 3). Hence bias for large *μ*_*C*_ is due to the use of the IV weights and/or to transformation bias, as restricted-range effects are not involved. Use of the bias-corrected estimator $\hat {\lambda }^{\Delta }$ does not affect bias in the IV-weighted estimators of *λ*. SSW, however, often produces aberrantly large values, because of the presence of $\bar {X}_{iT}^{2}$ and $\bar {X}_{iC}^{2}$ in the denominators in Eq. (2). An $\bar {X}_{iC}^{2}$ near zero considerably inflates a study’s $\hat {\lambda }_{i}$, producing an inflated SSW estimate of *λ*. Because the corresponding $\hat {v}_{i}^{2}$ (Eq. (3)) are also inflated, the IV-weighted methods downweight these studies, but SSW does not. To summarize, we do not recommend bias-correction under normal distribution.

Astonishingly, the MSE in estimation of *λ* does not reflect the biases of the respective estimators, but strongly depends on *K*, even though the bias in estimation of *λ* does not. The MSE of SSW is somewhat higher than MSE of the IV-weighted estimators when *K*=5, can be higher or lower when *K*=10 and is usually lower when *K*=30.

**Coverage** When *μ*_*C*_=1, coverage of all IV-weighted methods is considerably below nominal for *n*≤40 (with some exceptions near *τ*^2^=0 when *λ* is small) (Fig. 5, row 1). Coverage of the IV-weighted methods deteriorates substantially for larger *K* and larger values of *τ*^2^, especially for small values of *λ* (Fig. 5, rows 2 and 3). The HKSJ intervals provide nominal coverage only for *n*≥100, and the standard IV-weighted methods never achieve the nominal level. SSW MP, with *t* critical values, is the only choice, but its coverage is somewhat above nominal for very small *n*. When *μ*_*C*_=4, the HKSJ intervals provide nominal coverage starting from *n*=10 when *K*≤30 and *τ*^2^>0, and the coverage of all other IV-weighted methods is considerably lower than nominal. Coverage of all except SSW MP deteriorates for larger *K* (Fig. 5, row 4), though coverage of SSW MP is above nominal at *τ*^2^=0. Coverage of all methods except DL and J improves for very large sample sizes, but it still can be extremely low at zero. Use of the bias-corrected estimator $\hat {\lambda }^{\Delta }$ does not affect coverage of *λ*.

### Bias and coverage in estimation of *τ*^2^ under lognormal distribution (Figs. 6 and 7)

**Bias** When *n* is very small (Fig. 6, row 1), all four estimators of *τ*^2^ have substantial positive bias, increasing linearly with *τ*^2^ (when *λ*=0 and *n*=4, the intercept is around 0.4, and the slope is around 0.9). This pattern persists for *λ*≤1; but when *λ*=2, the slope is essentially 0 (Fig. 6, row 3). As *n* increases to 40, the intercept and slope decrease; but the trace for DL begins to diverge from the others, followed by the trace for J, and increasingly as *λ* increases. *K* has little effect. MP and REML have similar, reasonably small, bias when *n*≥40 (Fig. 6, rows 2 and 4). When *n*≥100, the traces for DL and J bend toward increasingly negative bias as *τ*^2^ increases; their bias becomes worse as *n* increases and slightly worse as *K* increases (for example, when *λ*=0,*n*=1000, and *K*≥50, the bias of DL is −0.28 at *τ*^2^=1). The bias correction for $\hat {\lambda }_{i}$ does not reduce the bias [33] (Appendices A2, A4, C2, and C4).

**Coverage** When *n*<40 and *K*=5, the coverage of all four intervals for *τ*^2^ is below the nominal 95%, especially when *n*=4 and *τ*^2^<0.4; increasing *K* to 10 and 30 reduces coverage substantially and makes this pattern worse (Fig. 7, row 1), and increasing *λ* has little effect. Increasing *K* to 50 and beyond reduces coverage further, even to 0 when *n*=4 and *τ*^2^=0 (Fig. 7, row 2). When *n*≥40 and *K*=5 or 10, BJ and J generally provide nominal or slightly higher coverage, and QP and PL are slightly lower. Situations with *K*≥30 are often quite challenging; BJ has low coverage for *K*≥30, and for larger *n* and *K*, coverage of J deteriorates similarly to BJ, but QP and PL provide good coverage (Fig. 7, row 3). Coverage of all methods improves for larger *λ* (Fig. 7, row 4). The bias correction does not improve coverage.

### Bias, mean squared error, and coverage in estimation of *λ* under lognormal distribution (Figs. 8, 9 and 10)

**Bias and mean squared error** All five estimators of *λ* have bias that shows little dependence on *K*. When *λ*=0 and *τ*^2^=0, they all have essentially no bias. When *τ*^2^>0, the bias is very roughly linear in *τ*^2^, with negative slope but a non-negative intercept for the IV-weighted estimators and a negative intercept for SSW. The intercept for the IV-weighted estimators is positive for *n*≥10, so their bias is positive for smaller *τ*^2^ and negative for larger *τ*^2^; but the traces flatten as *n* increases, and by *n*=40 their bias is positive for 0.1≤*τ*^2^≤1. The trace for SSW flattens similarly; and when *n*=40, its bias has smaller magnitude than the IV-weighted estimators when 0.1≤*τ*^2^≤0.5 and larger magnitude when 0.6≤*τ*^2^≤1 (see Fig. 8, rows 1 and 2). When *λ*>0, the biases of all five estimators at *τ*^2^=0 and the intercepts (i.e., biases) at *τ*^2^=0.1 increase; for a given *λ* both the intercepts and the slopes decrease as *n* increases. As a result, when *λ*≥0,5, the bias of SSW usually has smaller magnitude than the IV-weighted estimators. In relative terms, when *n*<40, the biases are substantial: as much as 10% of *λ* in some cases. Here SSW has the least bias, about 10% for *λ*≥1 and *n*=10, declining to 5% for *λ*≥1 and *n*=20 (Fig. 8, row 3). The bias correction for $\hat {\lambda }_{i}$ reduces the bias (Fig. 8, row 4) and should be used.

All estimators have approximately the same MSE. This may seem astonishing for row 1, where SSW is, on average, more biased than the IV-weighted estimators. The explanation lies in inefficiency of the IV weights based on the sample means and variances, for the lognormal distribution. The fixed weights are more efficient, and this is another argument for using SSW.

**Coverage.***t*-intervals centered at SSW provide the best coverage of *λ*, and that coverage is satisfactory when *n*≥20 and *K*≤30. Those intervals may have coverage greater than 97% (primarily when *τ*^2^=0 and *K*=5 or 10 and in a few cases where *τ*^2^=0.1,*K*=5,*n*=20 or 40, and *λ*≤0.5) or coverage less than 93% (mainly when *τ*^2^ is small, *K*≥50,*n*=20 or 40, and *λ*≥0.5). All other methods have inferior coverage and are not recommended (Fig. 10, row 1). Coverage of the intervals centered at SSW is better when the bias correction is used for $\hat {\lambda }_{i}$; then it is good when *n*≥10. When *n* is small, *K*≥50, and *λ*=0, coverage of the standard methods improves somewhat, whereas coverage of SSW MP becomes less than 93% when *K*>50, especially for large *τ*^2^ (Fig. 10, row 2). When the bias correction is used, coverage of SSW MP is the best, and it is good overall for small *λ*, but it is much below 95% for *n*=4,*λ*≥0.5 and small *τ*^2^, where it worsens for larger *K*. For large *K* and large *n*, coverage of SSW MP is still the best, especially at *τ*^2^=0, and the bias correction still produces better results (Fig. 10, rows 3 and 4).

### Examples

Two examples from the literature illustrate ways in which meta-analyses of LRR can differ. In the first example the standardized sample means appear to be normally distributed, whereas in the second example they are skewed to the right, so the individual-level data may be more likely to have come from lognormal distributions.

#### Example 1: mate choice copying

Mate choice copying (MCC, initially observed in various non-human animal species) occurs when an individual’s likelihood of accepting or choosing a potential mate is greater if others regard that mate as attractive. In controlled experiments, human subjects are commonly shown images of model individuals and asked to rate the model’s desirability, interest, or physical attractiveness. Designs differ between female and male subjects. Gouda-Vossos et al. [35] report that “The majority of studies on MCC effects in humans have focused on ‘individual-based copying’ where the dependent variable is the response of the subject toward the target individual presented alongside an opposite-sex other, compared with a target presented alone. Recent studies have suggested that MCC may … include assessing the underlying biological and social qualities of potential mates. For instance, a man in the presence of a ‘high quality woman of higher mate value’ who is physically attractive or has a desirable personality informs female copiers that the man must also have high-quality features that are not readily observable.”

**Fig. 11:**
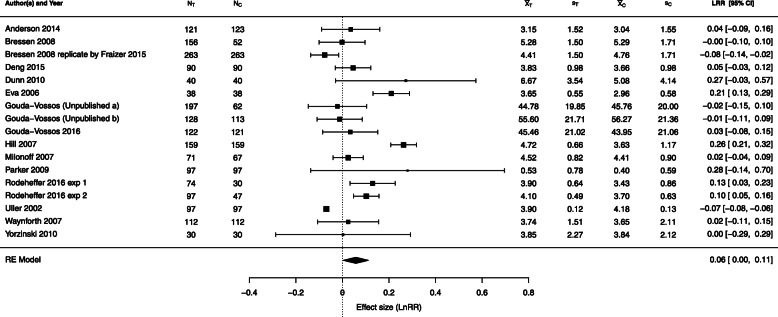
Forest plot for the meta-analysis of the effect of the addition of a female cue on female choice by Gouda-Vossos et al. [35] Subscripts *T* and *C* correspond to the arms with/without a female cue (i.e., the presence of a female). REML was used in estimating the between-study variance

In one meta-analysis Gouda-Vossos et al. combined data from 17 studies of female choice in which the experimental manipulation was the addition of a female cue; that is, the female subject viewed images of male targets presented with (vs. without) a female. The data are available from the supplementary material in Gouda-Vossos et al. [35] and are reproduced in Fig. 11. The arm-level sample sizes range from 30 to 263, and all but four studies have balanced sample sizes. The response ratio was used as the measure of effect size; a positive log-response-ratio indicates higher ratings when the male is in the presence of a female. Parker 2009 has substantially lower arm-level means than the other studies; but all values of $\sqrt {n_{ij}} \bar {X}_{ij} / s_{ij}$ are above 6, and only six are less than 12, so proximity to zero should not cause problems. Figure 11 shows the forest plot from the REML-based analysis. The authors concluded that “The positive mean revealed that men are rated 6.01% more attractive/desirable when in the presence of a female.” Q–Q plots of the standardized sample means (Additional File 2) showed their distribution to be approximately normal, though with some outliers. Therefore our simulation results for the normal distribution may be more relevant.

Table 2 shows the results from various methods of estimation. The estimators of *τ*^2^ show clear differences. DL provides the highest value at 0.0158, followed by J at 0.0119, REML at 0.0091, and MP at 0.0080, half the size of DL.

**Table 2:** Point and confidence-interval estimates for *τ*^2^ and *λ* in meta-analysis of the effect of the addition of a female cue on female choice; FE is fixed-effect model, and RE is random-effects model. The heterogeneity parameter is *τ*^2^. *L* and *U* denote the lower and upper limits of the 95% confidence intervals

Model	Method	$\hat {\tau }^{2}$	*L*	*U*	$\hat {\lambda }$	*L*	*U*	Length of CI
FE	IV	0			−0.0478	−0.0560	−0.0396	0.0164
RE	DL, QP	0.0158	0.0037	0.0230	0.0596	−0.0076	0.1268	0.1344
RE	BJ		0.0050	0.0671				
RE	J	0.0119	0.0046	0.0375	0.0585	−0.0012	0.1181	0.1193
RE	REML, PL	0.0091	0.0036	0.0275	0.0575	0.0041	0.1109	0.1068
RE	MP	0.0080			0.0571	0.0062	0.1080	0.1018
RE	$\hat {\lambda }^{\Delta }$ & MP	0.0080			0.0567	0.0059	0.1076	0.1017
RE	HKSJ (DL)				0.0596	0.0040	0.1151	0.1111
RE	HKSJ MP				0.0571	0.0020	0.1122	0.1102
RE	$\hat {\lambda }^{\Delta }$ & HKSJ MP				0.0567	0.0018	0.1117	0.1099
RE	SSW, SSW MP				0.0528	−0.0122	0.1178	0.1300
RE	$\hat {\lambda }^{\Delta }$ & SSW, SSW MP				0.0525	−0.0124	0.1173	0.1297

The closest simulation scenarios are for *λ*=0,*τ*^2^=0 and 0.1, and *n*=40 and *n*=100. Under these conditions for both normal and lognormal distributions, all methods overestimate *τ*^2^ at zero, but we do not have results for positive values of *τ*^2^ below 0.1. Our results show that for small *τ*^2^>0, the standard methods overestimate *λ*=0, and only SSW provides nearly unbiased estimates of *λ* under both normal and lognormal distributions. These results agree with our findings for this example. The SSW estimate of *λ*, 0.0528, is lower than all IV-weighted estimates (except FE, which is negative).

As the sample sizes are moderate to large, the differences in estimated *τ*^2^ have considerable effect on the width of the confidence intervals for *λ*.

Among the IV-weighted methods, only REML and MP produced confidence intervals for *λ* that did not include 0. These reflect the combination of a positively biased estimate of *λ* and the low estimate of *τ*^2^. The original meta-analysis used REML. Interestingly, the HKSJ confidence intervals based on MP and DL also do not cross 0. For HKSJ (DL), this happens because it is actually shorter than the DL interval for these data. The SSW MP CI is wider than the HKSJ confidence intervals, and it includes 0.

As the sample sizes are moderate to large, the bias correction is not needed. It is not recommended for normally distributed data in any case.

Because, in our simulations, the IV-weighted methods tend to give positively biased results, we consider the SSW point estimate to be close to the true value of *λ*, and the HKSJ MP interval to provide correct coverage. Therefore, we do not consider the effect of MCC in women to be significant.

#### Example 2: low-dose dopamine

Controversy surrounded the use of low-dose dopamine (a catecholamine with dose-dependent effects) to promote kidney function in several categories of patients (e.g., having various types of surgery, receiving intravenous contrast dye). In a systematic review and meta-analysis of randomized controlled trials that compared low-dose dopamine (≤5*μ*g/kg of body weight per minute) with placebo or no therapy, Friedrich et al. [36] examined clinical outcomes (mortality, need for renal replacement therapy, and adverse events) and renal outcomes (urine output, creatinine level, and creatinine clearance—each on Days 1, 2, and 3 after starting therapy). Because of differences among trials in the units for reporting urine output, Friedrich et al. could not use mean difference as the measure of effect. As a more clinically meaningful summary of treatment than the SMD, they chose the relative change in the dopamine group compared with the control group, analyzed as the log-transformed RoM.

**Fig. 12:**
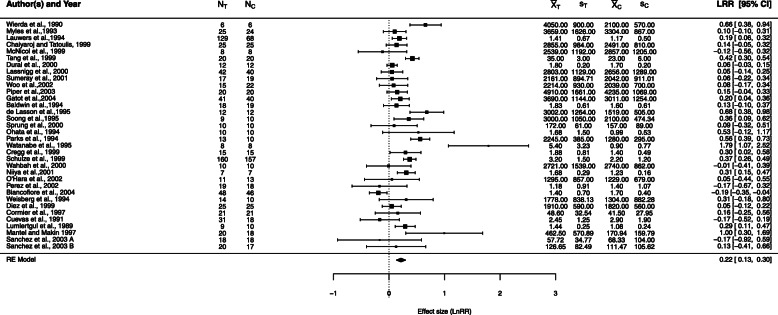
Forest plot for the meta-analysis on the effects of low-dose dopamine compared with placebo or no therapy (data provided by J. Friedrich). Subscripts *T* and *C* correspond to the arms with/without dopamine. DL was used in estimating the between-study variance. The studies reported urine output in a variety of units. For example, Baldwin 1994 used mL/kg, Sprung 2000 used mL/h, Cregg 1999 used ml/kg/h, and O’Hara 2002 used ml/24h

J. Friedrich kindly provided the data on the effect of low-dose dopamine therapy on urine output at Day 1 after its initiation, which we show in Fig. 12. We re-analyze the data from the full sample of 34 studies, with arm-level sample sizes ranging from 6 to 160 and, separately, the data from the 10 studies in the “Other surgery” category. All but two studies had balanced sample sizes. A positive log-response-ratio indicates higher urine output with dopamine. Because urine output is nonnegative, it is reasonable to assume that all *μ*_*ij*_>0. All $\bar {X}_{ij}$ are large enough that proximity to 0 should not be a problem. Figure 12 shows the forest plot from the DL-based analysis. The authors concluded that, on average, dopamine increased Day 1 urine output by 24%. Q–Q plots of the standardized sample means (Additional File 2) showed their distribution to be skewed to the right, so our simulation results for the lognormal distribution may be more relevant.

Table 3 shows the results from various methods of estimation. There are clear differences between the estimators of *τ*^2^. DL provides the lowest value at 0.038, followed by REML at 0.041, J at 0.057, and MP at 0.067, almost twice as large as DL. Bias correction results in lower estimates of *τ*^2^, from 0.032 for DL to 0.046 for J. The RE estimates of *λ* vary from 0.210 for SSW to 0.220 for MP. Bias correction further reduces the differences among RE estimates of *λ* (0.209 to 0.214). In both analyses, all methods find a significant effect of dopamine, with SSW MP providing the widest confidence intervals.

**Table 3:** Point and confidence-interval estimates for *τ*^2^ and *λ* in fixed-effect (FE) and random-effects meta-analysis of the effect of low-dose dopamine on Day 1 urine output, compared with placebo or no therapy. The heterogeneity parameter is *τ*^2^. *L* and *U* denote the lower and upper limits of the 95% confidence intervals

Method	*τ*^2^	$\tau _{L}^{2}$	$\tau _{U}^{2}$	$\hat {\lambda }$	*L*	*U*	$\exp (\hat {\lambda })$	*L*	*U*
All 34 studies									
FE	0			0.207	0.172	0.243	1.230	1.188	1.275
DL, QP	0.038	0.029	0.164	0.216	0.133	0.298	1.241	1.143	1.348
BJ		0.018	0.089						
J	0.057	0.030	0.118	0.219	0.123	0.315	1.244	1.130	1.370
REML, PL	0.041	0.018	0.094	0.216	0.131	0.301	1.241	1.140	1.351
MP	0.067			0.220	0.118	0.322	1.246	1.125	1.380
HKSJ (DL)				0.216	0.116	0.315	1.241	1.123	1.371
HKSJ MP				0.220	0.114	0.326	1.246	1.120	1.386
SSW, SSW MP				0.210	0.061	0.359	1.234	1.063	1.432
$\hat {\lambda }^{\Delta }$ & FE	0			0.196	0.153	0.240	1.217	1.165	1.271
$\hat {\lambda }^{\Delta }$ & DL, QP	0.032	0.015	0.126	0.212	0.128	0.295	1.236	1.137	1.344
$\hat {\lambda }^{\Delta }$ & BJ		0.014	0.081						
$\hat {\lambda }^{\Delta }$ & J	0.046	0.019	0.105	0.214	0.120	0.308	1.238	1.127	1.360
$\hat {\lambda }^{\Delta }$ & REML, PL	0.029	0.011	0.071	0.211	0.130	0.292	1.235	1.139	1.339
$\hat {\lambda }^{\Delta }$ & MP	0.043			0.213	0.121	0.306	1.238	1.129	1.357
$\hat {\lambda }^{\Delta }$ & HKSJ (DL)				0.212	0.119	0.304	1.236	1.126	1.356
$\hat {\lambda }^{\Delta }$ & HKSJ MP				0.213	0.118	0.309	1.238	1.125	1.362
$\hat {\lambda }^{\Delta }$ & SSW, SSW MP				0.209	0.079	0.338	1.232	1.082	1.402
10 studies in Other surgery									
FE	0			0.273	0.206	0.339	1.314	1.229	1.404
DL, QP	0.093	0.064	0.984	0.291	0.070	0.511	1.337	1.073	1.667
BJ		0.030	0.528						
J	0.146	0.054	0.611	0.303	0.038	0.567	1.353	1.039	1.764
REML, PL	0.161	0.038	0.672	0.305	0.029	0.582	1.357	1.029	1.789
MP	0.226			0.314	−0.005	0.633	1.369	0.995	1.884
HKSJ (DL)				0.291	−0.045	0.627	1.337	0.956	1.872
HKSJ MP				0.314	−0.054	0.682	1.369	0.947	1.979
SSW, SSW MP				0.270	−0.347	0.888	1.310	0.706	2.430
$\hat {\lambda }^{\Delta }$ & FE	0			0.244	0.164	0.324	1.277	1.178	1.383
$\hat {\lambda }^{\Delta }$ & DL, QP	0.088	0.034	0.895	0.275	0.050	0.501	1.317	1.051	1.650
$\hat {\lambda }^{\Delta }$ & BJ		0.026	0.518						
$\hat {\lambda }^{\Delta }$ & J	0.128	0.041	0.568	0.285	0.025	0.544	1.329	1.025	1.724
$\hat {\lambda }^{\Delta }$ & REML, PL	0.091	0.020	0.473	0.276	0.048	0.505	1.318	1.049	1.656
$\hat {\lambda }^{\Delta }$ & MP	0.166			0.292	0.003	0.580	1.339	1.003	1.787
$\hat {\lambda }^{\Delta }$ & HKSJ (DL)				0.275	−0.032	0.582	1.317	0.969	1.790
$\hat {\lambda }^{\Delta }$ & HKSJ MP				0.292	−0.041	0.625	1.339	0.960	1.868
$\hat {\lambda }^{\Delta }$ & SSW, SSW MP				0.271	−0.266	0.808	1.312	0.767	2.243

The closest simulation scenarios are for *λ*=0.2,*K*=30,*τ*^2^ between 0 and 0.1, and *n*=10 to *n*=100. Under these conditions for both normal and lognormal distributions, all methods overestimate *τ*^2^ when *n*=10, but are almost unbiased for *n*=40. We expect that all methods would provide coverage of *τ*^2^ that is too low, with QP being the best. We do not expect the bias correction to improve bias or coverage. Our results show that *λ*=0.2 is overestimated by the standard methods for small *τ*^2^>0, and only SSW provides nearly unbiased estimates of *λ* for both normal and lognormal underlying distributions. This agrees with our findings for this example. The SSW estimate of *λ*, 0.210 (or 0.209 after bias correction), is lower than all IV-weighted estimates.

For the 10 studies in the “Other surgery” subset, estimates of *τ*^2^ are higher, at 0.093 for DL to 0.226 for MP. From our simulations, they may be more positively biased, and in these data bias correction reduces estimates of *τ*^2^ to 0.088 to 0.166, respectively, probably reducing biases. We expect SSW to provide a nearly unbiased estimate of *λ*, at 0.270, with other methods positively biased (the highest at 0.314 for MP). Bias correction does not much affect the SSW estimate, but it does somewhat reduce the positive bias of the other methods (0.292 for MP). Coverage of *λ* should be nearly nominal for SSW MP (−0.347, 0.888) and lower than nominal for HKSJ MP, with the IV-weighted methods providing considerably lower coverage. In summary, the IV-weighted methods seem to indicate a significant effect of dopamine, whereas SSW MP does not.

## Discussion

Our simulations raise concern about the current state of meta-analysis of LRR. For that effect measure and some others, the relation between the studies’ estimated effects and their estimated variances has several undesirable results: the performance of all inverse-variance-weighted methods depends on the effect sizes, estimates of overall effects are biased, and their confidence intervals have below-nominal coverage, especially for small sample sizes. Our simulations show this clearly.

It is well known that the distribution of a ratio of normal random variables *X*/*Y* is, in general, heavy-tailed and has no moments. It can be approximated by a normal distribution only when both *X* and *Y* are independent and have positive means, and their coefficients of variation fulfill some conditions [37]. The log transformation is used to resolve this difficulty and to make the distribution of LRR closer to normality. Similarly, for a log-normal underlying distribution, the log transformation will make the LRR ‘almost normal’. However, as we have seen, the LRR remains a very challenging effect measure.

For normal underlying distributions, we found considerable biases in all methods of estimating *τ*^2^ for very small *n*, but overall MP and REML are reasonable choices, especially for *τ*^2^ farther from zero. QP provides reasonable, though not perfect, coverage. For lognormal underlying distributions, *τ*^2^ cannot be estimated reliably for sample sizes smaller than 100; but once more, MP is a good choice for larger sample sizes.

Most applications of meta-analysis aim primarily to provide point and interval estimates of an overall effect. For general use, a point estimator should be unbiased, and a confidence interval should have (close to) nominal coverage.

After estimating the between-study variance *τ*^2^, customary practice uses an inverse-variance-weighted mean to estimate the overall effect. The origin of the IV-weighted approach lies in the fact that, for known variances, and given unbiased estimates of the study-level effects, it provides a uniformly minimum-variance unbiased estimate (UMVUE). However, in practice, the within-study variances are unknown, and using estimates for them leads to bias in the IV-weighted estimate of the overall effect and below-nominal coverage of the confidence interval. Thus, the IV-weighted approach is misguided; for most measures of effect, it cannot avoid these shortcomings.

To improve the coverage of confidence intervals systematically, we would like to have a better understanding of the underlying sampling distributions. For the IV-weighted estimators of the overall effect, in this study, we have retained the customary use of critical values from the normal distribution. To remedy the familiar problem of below-nominal coverage, the HKSJ interval uses the *t* distribution on *K*−1 degrees of freedom, and the SSW MP interval adopts that choice. We have, however, seen no empirical evidence relating these choices of reference distribution to the sampling distributions of the point estimators, for LRR or other measures of effect. In future work we intend to investigate sampling distributions of estimators of overall effect and *τ*^2^.

A reviewer called attention to a modification of the IV-weighted estimator of the overall effect that takes into account the relative quality of the studies [38, 39]. That approach has not been studied much in simulations, perhaps because of the need to assign judgments of the probability that each study is credible.

The gaps in evidence include the possibility that the unit-level variances in the two arms may differ, which simulations rarely, if ever, reflect. Because of the extent of our simulations, we did not attempt to fill this gap. However, we do not expect the performance of the IV-weighted methods to improve under more-challenging scenarios. Rather they are likely to perform worst in situations where the (within-study) variances of *X*_*iT*_ and *X*_*iC*_ differ substantially.

A pragmatic solution to unbiased estimation of *λ* uses weights that do not involve estimated variances (for example, weights proportional to the studies’ sample sizes *n*_*i*_). Our point estimator SSW uses weights proportional to an effective sample size, $\tilde {n}_{i} = n_{iC}n_{iT}/n_{i}$. Then the estimate of the overall effect is $\hat {\lambda }_{\mathit {SSW}}=\sum \tilde {n}_{i}\hat {\lambda }_{i}/\sum \tilde {n}_{i}$, and the estimate of its variance comes from Eq. (7). Finally, the *t*-based confidence interval for *λ* is centered at $\hat {\lambda }_{\mathit {SSW}}$.

For underlying lognormal distributions, SSW, combined with the bias-corrected estimator of *λ*_*i*_, works reasonably well for sample sizes as low as 10 in interval estimation of *λ*, and for *n*≥40 in point estimation. We recommend this method for further use in applications.

Unfortunately, for underlying normal distributions, the use of sample-size-based weights does not avoid all the problems with bias in estimation of *λ* for very small sample sizes and *λ* close to 0, and the bias correction is harmful. The response ratio is a rather challenging effect measure, with two additional sources of possible bias: its restricted range near 0 under the normal model, and transformation bias in using the log scale for specification of random effects. Both issues are inherent in the choice of the respective true model and cannot be easily resolved. Farther from 0, SSW is practically unbiased, and we recommend its use, in combination with SSW MP, which is the only feasible option for confidence intervals.

## Conclusions

- In extensive simulations the four point estimators of *τ*^2^ that we included all had considerable negative bias for normal within-study data and very small study-level sample sizes (*n*), especially when one of the means was close to zero. For lognormal underlying data, *τ*^2^ cannot be estimated reliably for *n*<40. MP had the least bias overall, and DL should not be used.
- For normal underlying data, the four interval estimators of *τ*^2^ that we included had coverage that often was below the nominal 95%, in various patterns, especially as the number of studies (*K*) increased. For lognormal underlying data, many situations were quite challenging. The QP and, to a lesser extent, PL interval estimators had coverage close to 95% when *n*≥100 or when *n* was moderate and *K* was small.
- Point estimators of *λ* that use inverse-variance weights had substantial bias, systematically related to *τ*^2^, for normal underlying data, but less bias (with weaker relation to *τ*^2^) for lognormal data.
- An estimator whose weights used only arm-level sample sizes (SSW) typically has less bias and is recommended. Additionally, we recommend bias correction for lognormal data, but it is contraindicated for normal data.
- We recommend an interval estimator based on SSW. It mostly had coverage close to 95% for both normal and lognormal data, but for small sample sizes (*n*≤40) and large numbers of studies (*K*≥50) its coverage was sometimes low. Interval estimators based on the inverse-variance-weighted point estimators were inferior, in various patterns, and we do not recommend them.

## Supplementary information

**Additional file 1** Q-Q plots for the standardized means in Examples 1 and 2.

**Additional file 2** R procedures to implement MP SSW, HKSJ MP, and MP IV methods for meta-analysis of LRR.

**Additional file 3** Relation of I^2^ to parameters underlying our simulations.

## Data Availability

Our full simulation results are available as e-prints (Bakbergenuly et al.[32] and Bakbergenuly et al. [33]). An R procedure to implement the MP SSW, HKSJ MP, and MP IV methods for meta-analysis of LRR is provided in Additional File 1.

## Abbreviations

BJ: Biggerstaff and Jackson interval
CV: Coefficient of variation
DL: DerSimonian-Laird
FE: Fixed effect
HKSJ: Hartung-Knapp-Sidik-Jonkman interval
IV: Inverse-variance
J: Jackson estimator/interval
LOR: Log-odds-ratio
LRR: Log-response-ratio
MP: Mandel-Paule estimator
MSE: Mean squared error
PL: Profile likelihood
QP: Q-profile interval
Q-Q plot: Quantile-quantile plot
RE: Random effects
REML: Restricted maximum likelihood
RoM: Ratio of means
RR: Response ratio
SMD: Standardized mean difference
SSW: Sample-size-weighted estimator
UMVUE: Uniformly-minimum-variance-unbiased estimator
