# Supplementary material for: Estimation in meta-analyses of response ratios
Source: BMC Med Res Methodol. 2020 Oct 22;20:263. doi: 10.1186/s12874-020-01137-1 (PMC7579974; doi:10.1186/s12874-020-01137-1)
Supplement: Supplementary file 1 — Additional file 1 Q-Q plots for the standardized means in Examples 1 and 2. [file 12874_2020_1137_MOESM1_ESM.pdf]

Additional Files

Additional File 1: QQ plots for the standardised means in Examples 1 and 2

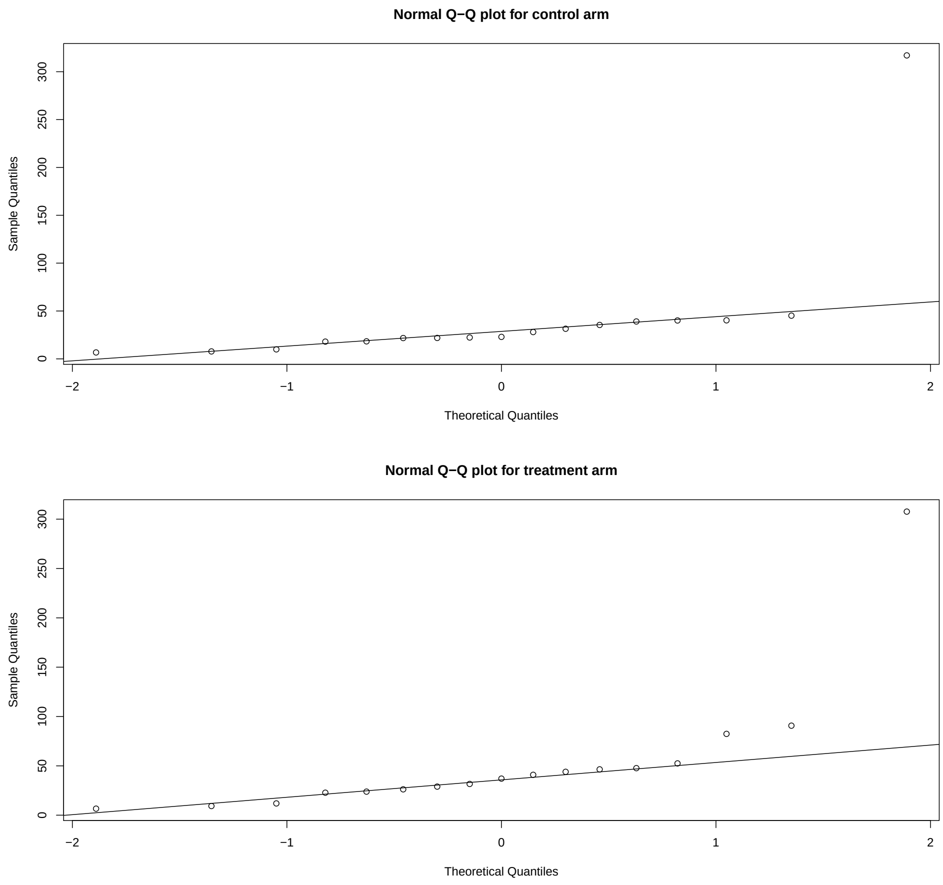

Figure A1: Normal QQ plots of the standardised means in the Treatment and Control arms for the meta-analysis of the effect of the addition of a female cue on female choice by Gouda-Vossos et al. (2018) (Example 1).

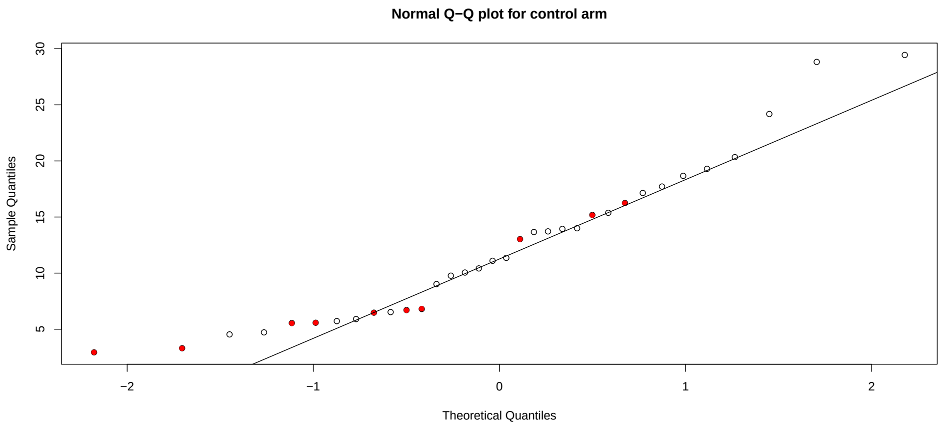

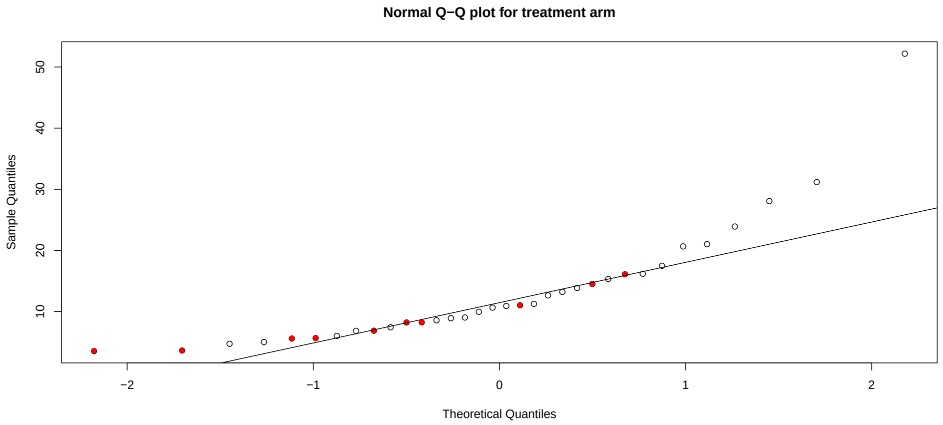

Figure A2: Normal QQ plots of the standardised means in the Treatment and Control arms for the meta-analysis on the effects of low-dose dopamine compared with placebo or no therapy (Example 2). Ten studies with "Other therapy" are in red.
