# Supplementary material for: Estimation in meta-analyses of response ratios
Source: BMC Med Res Methodol. 2020 Oct 22;20:263. doi: 10.1186/s12874-020-01137-1 (PMC7579974; doi:10.1186/s12874-020-01137-1)
Supplement: Supplementary file 2 — Additional file 2 R procedures to implement MP SSW, HKSJ MP, and MP IV methods for meta-analysis of LRR. [file 12874_2020_1137_MOESM2_ESM.pdf]

Additional File 2: R procedures to implement MP SSW, HKSJ MP, and MP IV methods for meta-analysis of LRR

A1.1 Example - Effect of the addition of a female cue on female choice

```
xT<-c(3.1500,  5.2800, 4.4100,  3.8300,  6.6700, 3.6500,
    44.7800, 55.6000, 45.4600,  4.7200, 4.5200,  0.5269,
    3.9050,  4.1000,  3.9050,  3.7400,  3.8500)
xC<-c(3.0400,  5.2900, 4.7600,  3.6590,  5.0800, 2.9600,
    45.7600, 56.2700, 43.9500,  3.6300, 4.4100,  0.3983,
    3.4300,  3.7000,  4.1850,  3.6500,  3.8400)
sT<-c(1.5200,  1.5000,  1.5000, 0.9800,  3.5400,  0.5500,
    19.8500, 21.7100, 21.0200,  0.6560,  0.8200,  0.7827,
    0.6400,  0.4900,  0.1250,  1.5100,  2.2700)
sC<-c(1.5500,  1.7100,  1.7100, 0.9800,  4.1360,  0.5800,
    20.0000, 21.3600, 21.0600,  1.1720,  0.9000,  0.5859,
    0.8600,  0.6300,  0.1300,  2.1100,  2.1200)
nT<-c(121, 156, 263,  90,  40,  38, 197, 128, 122, 159,  71,
      97,  74,  97,  97, 112,  30)
nC<-c(123, 52, 263,  90,  40,  38,  62, 113, 121, 159, 67,
    97,  30,  47,  97, 112,  30)
################################################################
library(metafor)
dat <- escalc(measure="ROM",m1i=xT,sd1i=sT,n1i=nT,m2i=xC,
    sd2i=sC,n2i=nC)
yi<-as.numeric(dat$yi)
vi<-as.numeric(dat$vi)
#estimation of between-study variance using MP method
res1<-metaRoM(yi,vi)
res1
               estimate         ci.lb        ci.ub
tau2 (MP) 0.008035441 0.003691526 0.02301641
################################################################
#meta-analysis of log-response ratio by SSW MP method
res2<-metaSSWwithMP(yi,vi,nC,nT)
res2
                    estimate          ci.lb        ci.ub
tau2 (MP)         0.008035441  0.003691526 0.02301641
theta (SSW MP)  0.052791032  -0.012184748 0.11776681
################################################################
#meta-analysis of log-response ratio by HKSJ MP method
res3<-metaHKSJwithMP(yi,vi,nC,nT)
res3
                     estimate          ci.lb        ci.ub
tau2 (MP)          0.008035441 0.003691526 0.02301641
theta (HKSJ MP)  0.057092835 0.002007003 0.11217867
```

A1.2 R procedures to implement MP SSW, HKSJ MP, and MP IV methods

```
#function for estimation of between-study variance using
#Mandel-Paule method
metaRoM<-function(yi,vi){
ll<-0
uu<-1000
K0<-length(yi)
f<-function(g,sigma,theta,K){
sum((theta-sum(theta/(sigma+g))/sum(1/(sigma+g)))^2/
(sigma+g))-K+1}
if (f(ll,sigma=vi,theta=yi,K=K0)*f(uu,sigma=vi,theta=yi,K=K0
    )<0)
{
tau2MP<- as.numeric(uniroot(f, c(ll, uu), tol = 0.0001,
    sigma=vi,theta=yi,K=K0)[1])}
f1<-function(g,sigma,theta,K){
sum((theta-sum(theta/(sigma+g))/sum(1/(sigma+g)))^2/
(sigma+g))-qchisq(0.025,K-1)}
if (f1(ll,sigma=vi,theta=yi,K=K0)*f1(uu,sigma=vi,theta=yi,K=
    K0)<0)
{
tau2U<- as.numeric(uniroot(f1, c(ll, uu), tol = 0.0001,
    sigma=vi,theta=yi,K=K0)[1])}
f2<-function(g,sigma,theta,K){
sum((theta-sum(theta/(sigma+g))/sum(1/(sigma+g)))^2/
(sigma+g))-qchisq(0.975,K-1)}
if (f2(ll,sigma=vi,theta=yi,K=K0)*f2(uu,sigma=vi,theta=yi,K=
    K0)<0)
{
tau2L<-as.numeric(uniroot(f2, c(ll, uu), tol = 0.0001, sigma
    =vi,theta=yi,K=K0)[1])}
tau2 <- data.frame(tau2MP,tau2L,tau2U)
colnames(tau2) <- c("estimate", "ci.lb", "ci.ub")
rownames(tau2) <- c("tau2")
return(tau2)
}
##########################################################
#Function for calculation of overall effect measure
#based on SSW
#User needs to input values for effects yi,
#within-study variances vi,
#sample sizes nC and nT in control and treatment groups
#and any estimator of between study variance - tau2
#required functions:None
metaSSWwithMP <- function(yi,vi,nC,nT){
```

```
#estimation of between-study variance
tau2_estimates<-metaRoM(yi, vi)
tau2 <- tau2_estimates[[1]]
K <- length(yi)
n=nC+nT
nbar <- (nC*nT)/n
thetabar_SSW <- sum(yi*nbar)/sum(nbar)
varThetaBar_SSW <- sum((vi+tau2)*(nbar^2))/((sum(nbar))^2)
Ltheta_SSW <- thetabar_SSW-qt(.975, df=K-1)*sqrt(varThetaBar
    _SSW)
Utheta_SSW <- thetabar_SSW+qt(.975, df=K-1)*sqrt(varThetaBar
    _SSW)
theta_estimates=data.frame(thetabar_SSW, Ltheta_SSW, Utheta_
    SSW)
names(theta_estimates) <- names(tau2_estimates)
output <- rbind(tau2_estimates, theta_estimates)
colnames(output) <- c("estimate","ci.lb","ci.ub")
rownames(output) <- c("tau2 (MP)","theta (SSW_MP)")
return(output)
}
#########################################################
##Hartung-Knapp-Sidik-Jonkman (HKSJ) method
metaHKSJwithMP <- function(yi, vi, nC, nT){
#estimation of between-study variance
tau2_estimates<-metaRoM(yi, vi)
tau2 <- tau2_estimates[[1]]
K <- length(yi)
thetabar_HKSJ<-sum(yi/(vi+tau2))/sum(1/(vi+tau2))
w_HKSJ<-1/(vi+tau2)
var_HKSJ<-sum(w_HKSJ*(yi-thetabar_HKSJ)^2)/((K-1)*sum(w_HKSJ
    ))
Ltheta_HKSJ<-thetabar_HKSJ-qt(.975, df=K-1)*sqrt(var_HKSJ)
Utheta_HKSJ<-thetabar_HKSJ+qt(.975, df=K-1)*sqrt(var_HKSJ)
theta_estimates=data.frame(thetabar_HKSJ, Ltheta_HKSJ, Utheta_
    HKSJ)
names(theta_estimates) <- names(tau2_estimates)
output <- rbind(tau2_estimates, theta_estimates)
colnames(output) <- c("estimate","ci.lb","ci.ub")
rownames(output) <- c("tau2 (MP)","theta (HKSJ_MP)")
return(output)
}
#########################################################
#Standard inverse-variance method
metaIV <- function(yi, vi, nC, nT, tau2){
K <- length(yi)
thetabar_IV <- sum(yi/(vi+tau2))/sum(1/(vi+tau2))
```

```
w_IV <- 1/(vi+tau2)
Ltheta_IV <- thetabar_IV-qnorm(.975)/sqrt(sum(w_IV))
Utheta_IV <- thetabar_IV+qnorm(.975)/sqrt(sum(w_IV))
output=data.frame(thetabar_IV,Ltheta_IV,Utheta_IV)
colnames(output) <- c("estimate","ci.lb","ci.ub")
rownames(output) <- c("thetaIV")
return(output)
}

##########################################################
```
