# Supplementary material for: Estimation in meta-analyses of response ratios
Source: BMC Med Res Methodol. 2020 Oct 22;20:263. doi: 10.1186/s12874-020-01137-1 (PMC7579974; doi:10.1186/s12874-020-01137-1)
Supplement: Supplementary file 3 — Additional file 3 Relation of I2 to parameters underlying our simulations. [file 12874_2020_1137_MOESM3_ESM.pdf]

Additional File 3: Relation of I^2 to parameters underlying our simulations

We calculated the heterogeneity measure I^2 as

$$I^2 = 100\tau^2/(\tau^2 + s^2),$$

where s^2 is the average within-study variance.

Our simulations used equal sample sizes for the Treatment and Control arms, $n_T = n_C = n/2$, constant across all K studies. More generally, an additional parameter, q, can control the balance between n_T and n_C: $n_T = \lceil(1-q)n\rceil$ and $n_C = n - n_T$. The within-study variances, given by Equation (1), are also equal, $v_i^2 \equiv s^2$, when the true sample variances, σ_T^2 and σ_C^2, are substituted for the estimates s_{ij}^2 in Equation (1) and the true means, μ_T and μ_C, are substituted for $\bar{X}_T$ and $\bar{X}_C$, respectively. As a result the value of I^2 does not depend on the number of studies.

In general, then, I^2 depends on n, μ_C, $\lambda = \log(\mu_T/\mu_C)$, σ_T^2, σ_C^2, and, of course, τ^2. Throughout our simulations $\sigma_T^2 = \sigma_C^2 = 1$. The plots below include two values of q, $q = .5$ and $q = .75$.

Figures A3 and A4 show I^2 on the vertical axis and $\tau^2 = 0.1(0.1)1.0$ on the horizontal axis ($I^2 \equiv 0$ when $\tau^2 = 0$), for $\mu_C = 1$ and $\mu_C = 4$. Each plot corresponds to a value of q (= .5 or .75) and a value of n (= 20, 40, 100, or 250), with traces for $\lambda = 0$, 0.2, 0.5, 1, and 2.

The formula for I^2 given above corresponds to the definition in Higgins and Thompson (2002) and is easy to use in simulations, where τ^2 and s^2 are known. Applications, however, must use estimates, and the customary formula, based on Cochran's Q,

$$I^2 = 100 \times \max\left\{0, \frac{Q-(K-1)}{Q}\right\},$$

corresponds to defining the "typical" within-study variance as a weighted harmonic mean of the v_i^2 (Hoaglin, 2016). When the v_i^2 are equal and the weights are equal, the weighted harmonic mean equals the arithmetic mean, but this is not true in general.

References

Higgins, Julian and Thompson, Simon G.: Quantifying heterogeneity in a meta-analysis. Statistics in Medicine **21**(11), 1539–1558 (2002).

Hoaglin, David C.: Misunderstandings about Q and 'Cochran's Q test' in meta-analysis. Statistics in Medicine **35**(4), 485–495 (2016).

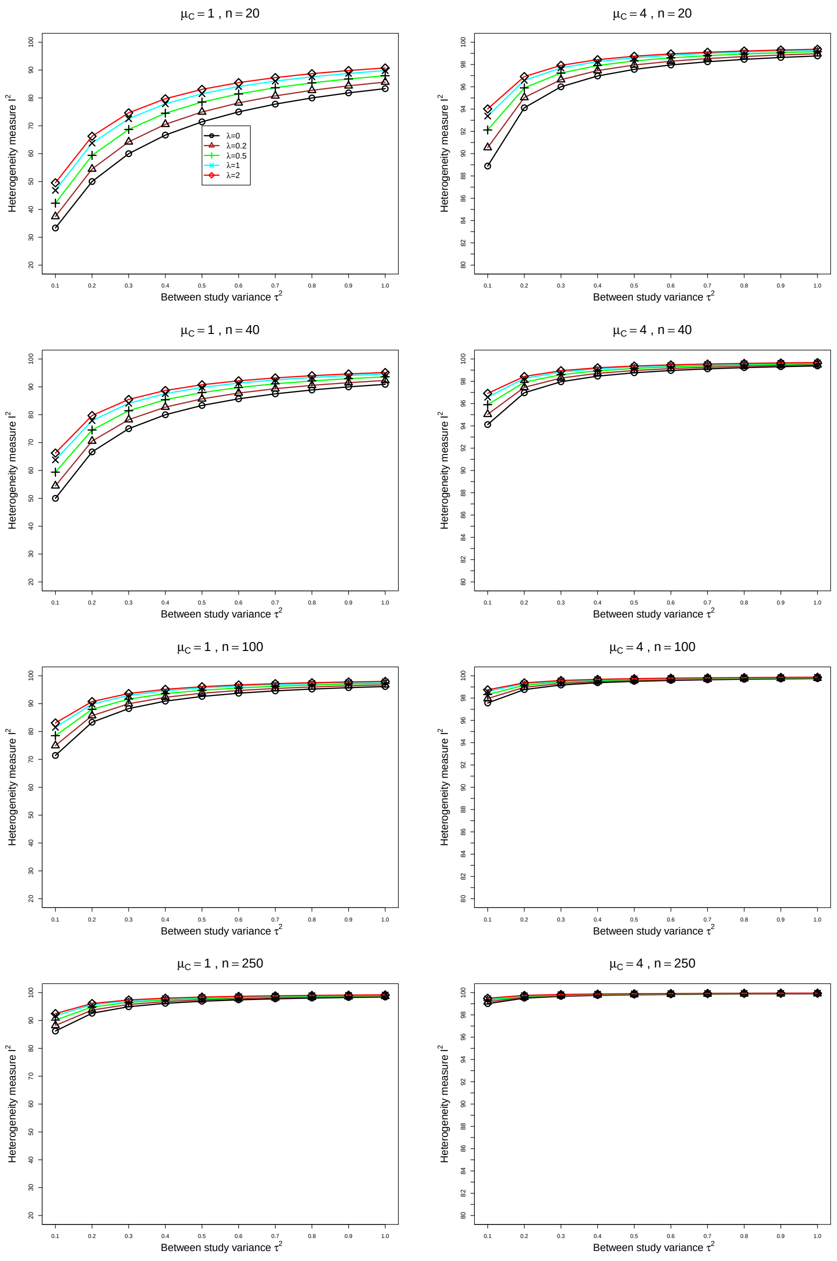

Figure A3: Heterogeneity measure I^2 versus $\tau^2 = 0.1(0.1)1.0$ for LRR when $\mu_C = 1$ and 4, $n = 20, 40, 100, 250$, and $q = .5$. The traces correspond to values of λ.

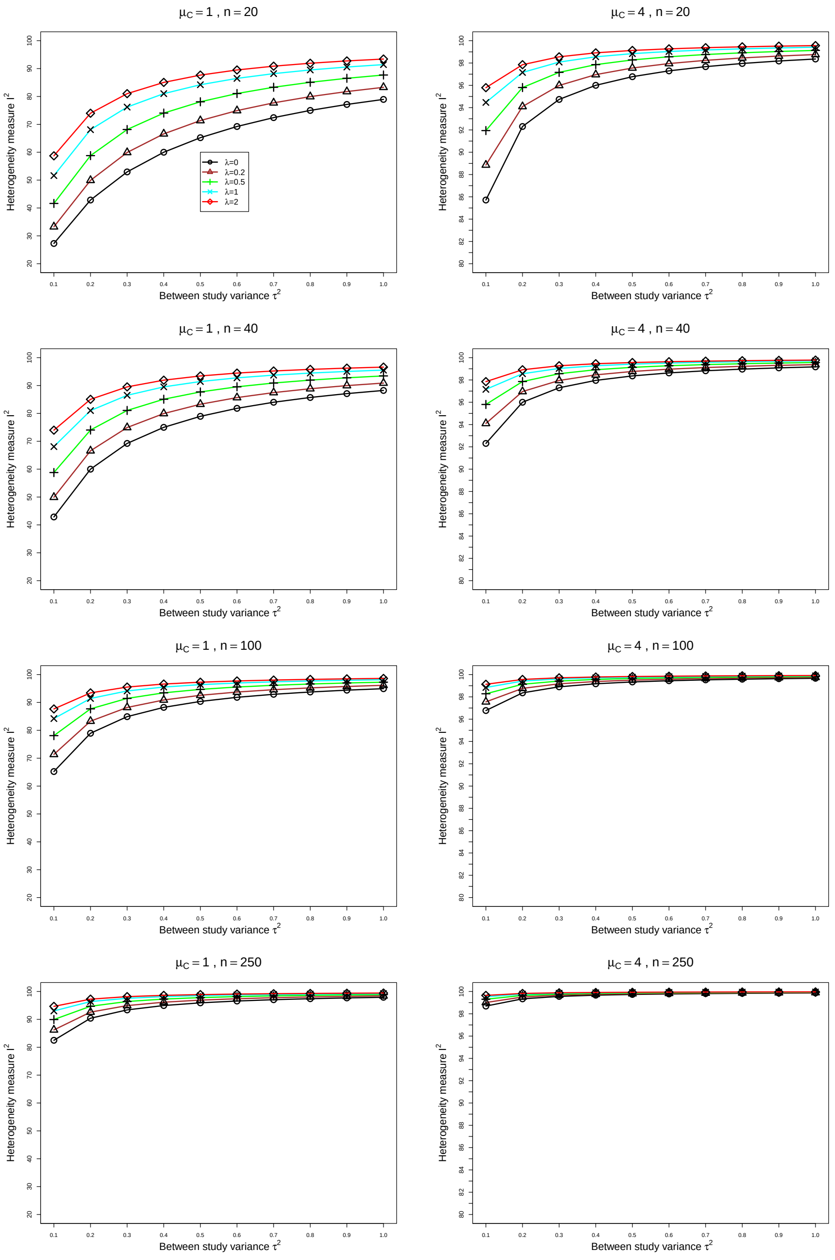

Figure A4: Heterogeneity measure I^2 versus $\tau^2 = 0.1(0.1)1.0$ for LRR when $\mu_C = 1$ and 4, $n = 20, 40, 100, 250$, and $q = .75$. The traces correspond to values of λ.
